# A narrative review of dietary patterns in cardiovascular-kidney-metabolic syndrome

**DOI:** 10.3389/fnut.2026.1799305

**Published:** 2026-07-14

**Authors:** Luo Lv, Yuli Guo, Xiaofang Li, Qinghua Han, Haixiong Wang

**Affiliations:** 1 Shanxi Medical University, Taiyuan, Shanxi, China; 2 First Clinical Medical College of Shanxi Medical University, Taiyuan, Shanxi, China; 3 Department of Digestive Oncology, Shanxi Bethune Hospital, Tongji Shanxi Hospital, Third Hospital of Shanxi Medical University, Taiyuan, Shanxi, China; 4 Department of Cardiology, The First Hospital of Shanxi Medical University, Taiyuan, China; 5 Department of Cardiology, Cardiovascular Hospital Affiliated to Shanxi Medical University, Shanxi Key Laboratory of Heart Failure Precision Medicine, Shanxi Cardiovascular Hospital (Institute), Shanxi Clinical Medical Research Center for Cardiovascular Disease, Taiyuan, Shanxi, China

**Keywords:** cardiovascular disease, chronic kidney disease, CKM syndrome, dietary patterns, type 2 diabetes mellitus

## Abstract

Driven by the global rise in obesity and lifestyle transitions, cardiovascular-kidney-metabolic (CKM) syndrome has emerged as a pathophysiological continuum characterized by metabolic dysregulation and involving multi-organ interactions. Within the comprehensive management of CKM syndrome, dietary patterns represent a cornerstone of intervention due to their high modifiability and cost-effectiveness. Adopting the perspective of the CKM syndrome pathophysiological continuum, this narrative review provides a thematic overview of the current literature on the Mediterranean, DASH, plant-based, and ketogenic diets in relation to metabolic syndrome, type 2 diabetes mellitus, chronic kidney disease, and cardiovascular disease. Evidence indicates that the Mediterranean and DASH diets, through established anti-inflammatory, antioxidant, and endothelial protective mechanisms, are the most consistently supported dietary patterns for CKM risk mitigation. The efficacy of plant-based diets is strictly quality-dependent: while healthful patterns rich in whole grains and vegetables are associated with improved cardiorenal outcomes, unhealthful patterns dominated by refined carbohydrates may exacerbate metabolic derangements. Although the ketogenic diet may improve glucose metabolism in the short term, concerns regarding elevated low-density lipoprotein cholesterol, potential hepatotoxicity, and limited long-term adherence suggest that its role may be more relevant in selected short-term settings than as a sustained long-term dietary pattern. Furthermore, structured dietary quality indices may provide useful tools for characterizing dietary exposure in relation to CKM and for generating mechanistic hypotheses. By integrating clinical and mechanistic evidence, this review outlines a stage-oriented conceptual framework to discuss how different dietary patterns may relate to distinct phases of the CKM syndrome.

## 1 Introduction

In recent years, driven by the accelerating global aging population and shifts in lifestyle, the prevalence of metabolic diseases, chronic kidney disease (CKD), and cardiovascular disease (CVD) has risen sharply (1, 2). Traditionally, clinical practice has treated these conditions as independent pathological entities. However, accumulating evidence now reveals complex pathophysiological interactions between metabolic abnormalities, impaired renal function, and cardiovascular dysfunction. In response to these findings, the American Heart Association (AHA) released a landmark Presidential Advisory in 2023, formally introducing the concept of “cardiovascular-kidney-metabolic (CKM) syndrome” (3). CKM syndrome not only defines a systemic spectrum of disease encompassing metabolic risk factors, CKD, and cardiovascular pathology, but it also emphasizes the critical importance of early screening and life-course management in improving patient outcomes.

Lifestyle intervention is considered the cornerstone of CKM syndrome prevention and treatment (3). Among these interventions, dietary patterns have gained significant attention as one of the most modifiable and cost-effective factors. Unlike single-nutrient studies, dietary pattern research focuses on the synergistic effects between foods and nutrients, offering a more holistic view of an individual's long-term dietary exposure. The Life Essential 8 framework of the AHA incorporates diet as a core metric, with other key components, such as blood glucose, lipids, and body mass index, being profoundly influenced by dietary habits (4). Recent evidence further supports that health behavior scores based on this framework are strongly associated with reduced incidence and mortality of CKM syndrome, reinforcing the importance of lifestyle interventions in improving patient prognosis (5).

Given this context, this narrative review summarizes the associations between major dietary patterns and CKM syndrome, focusing on its core pathophysiological components. From the perspective of the CKM continuum, we discuss the potential relevance of distinct dietary patterns across the spectrum of disease, from early metabolic dysregulation to advanced target organ damage. By integrating biological mechanisms with clinical evidence, this review aims to discuss the potential relevance of different dietary patterns across stages of the CKM syndrome.

## 2 Literature search and bibliometric analysis

### 2.1 Scope of the narrative review and literature search strategy

This review was conducted as a structured narrative review supported by bibliometric analysis and evidence mapping. PubMed and Web of Science were searched for the narrative literature review, while the bibliometric analysis was based on records retrieved from the Web of Science Core Collection. English-language publications published between 2010 and 2025 were searched using combinations of terms related to “cardiovascular–kidney–metabolic syndrome,” “Mediterranean diet,” “dietary approaches to stop hypertension diet,” “DASH diet,” “plant-based diet,” “ketogenic diet,” “metabolic syndrome,” “type 2 diabetes mellitus,” “chronic kidney disease,” and “cardiovascular disease.” In addition, the reference lists of relevant reviews and original studies were manually checked to identify additional publications of direct relevance.

Priority was given to randomized controlled trials, large prospective cohort studies, systematic reviews, meta-analyses, and mechanistic studies directly relevant to dietary patterns and CKM-related outcomes. The scope of this review was structured around four major dietary patterns: the Mediterranean diet (MedDiet), dietary approaches to stop hypertension (DASH) diet, plant-based diet (PBD), and ketogenic diet (KD). Evidence was then narratively summarized according to the major components of CKM syndrome, including metabolic syndrome (MetS), type 2 diabetes mellitus (T2DM), chronic kidney disease (CKD), and cardiovascular disease (CVD).

To enhance the objectivity and transparency of evidence synthesis, we constructed a structured evidence-mapping table summarizing the characteristics of the four major dietary patterns, their relevance to different stages of CKM syndrome, and potential mechanisms (Table 1). This evidence mapping, together with the bibliometric findings, informed the thematic organization of the review and helped reduce reliance on subjective classification.

**Table 1:** Evidence mapping of major dietary patterns and their potential relevance across stages of CKM syndrome.

Feature/diet pattern	MedDiet	DASH diet	PBD	KD
Characteristics	Plant-based foods and olive oil; moderate fish/dairy; limited red meat and refined sugars (37)	Fruits, vegetables, whole grains, low-fat dairy; low sodium; limited saturated fat and sugar (142)	Emphasizes plant sources, excludes or reduces animal products (categorized as hPDI/uPDI) (204)	Extremely low carb, high fat, moderate protein (262)
Representative clinical settings	Patients with multiple metabolic abnormalities, systemic inflammation, established cardiovascular risk, or those seeking a sustainable long-term dietary pattern (52–54, 77–81, 111–113, 131)	Patients with hypertension, MetS, or elevated cardiovascular risk (34, 142–144, 180, 185)	Patients with metabolic disorders or T2DM; mainly when diet quality is high (205–209, 212, 215, 222, 223, 225)	Patients with obesity or T2DM requiring short–term metabolic improvement (263, 265–269, 282, 283)
Primary metabolic associations (CKM Stage 1–2)	Associated with lower FBG, HbA1c, WC, body weight, and TG (52, 53, 77–81), and with lower risks of MetS (38, 39) and T2DM (65–67)	Associated with BP reduction (148, 149) and with lower FBG, WC, body weight, and TG (143, 144, 151–153)	hPDI: associated with lower MetS/T2DM risk; uPDI: associated with higher metabolic risk (205, 222, 223)	Associated with rapid reductions in blood glucose, body weight, and medication requirements (263, 268, 278, 283, 287)
Kidney-related associations (CKM stage 2–3)	Associated with lower CKD risk, slower eGFR decline, and less proteinuria (101–104)	Associated with lower CKD risk and with more stable renal function (100, 174, 175), although short-term effects on eGFR may be limited (102)	Mainly favorable for hPDI: associated with lower CKD risk and better eGFR (95, 236, 237)	Kidney-related data remain limited; potential benefit has been reported in DKD and ADPKD (293–296)
Cardiovascular associations (CKM stage 3–4)	Associated with lower CVD and MACE risk (111–113, 131)	Associated with lower HF risk; secondary prevention findings are mixed (185, 188–191)	Mainly favorable for hPDI: associated with lower CVD and mortality risk (248, 249)	Short-term metabolic improvement, but LDL-C may rise (307)
Primary mechanisms	Anti-inflammatory, antioxidant, endothelial protection, lipid profile improvement (58, 61, 87, 107, 121–125)	Sodium/potassium/magnesium balance, inhibits RAAS, vasodilation, anti-inflammatory (154, 155, 168)	Anti-inflammatory, improves gut microbiota, metabolic remodeling (218, 219, 221, 223, 231, 234, 243, 244, 259, 260)	Improving insulin sensitivity and gut microbiota (275, 276, 278, 279, 290, 291)
Safety/limitations	Heterogeneous across populations; influenced by adherence and cultural context (47–51)	Significant blood pressure reduction; heterogeneous clinical outcomes (148, 149)	Need to distinguish diet quality; wary of sarcopenia (243); monitor potassium in late CKD (potassium binders available) (241)	LDL-C elevation; liver toxicity (302); low long-term compliance (266)
Potential relevance to different stages of CKM syndrome	Stage 1–4	Stage 1–3: particularly in patients with elevated or poorly controlled blood pressure	Stage 1–4: mainly hPDI	Stage 1–2: particularly in short-term or intermittent settings

CKM, cardiovascular-kidney-metabolic; MedDiet, Mediterranean diet; DASH, dietary approaches to stop hypertension; PBD, plant-based diet; hPDI, healthy plant-based diet index; uPDI, unhealthy plant-based diet index; KD, ketogenic diet; MetS, metabolic syndrome; T2DM, type 2 diabetes mellitus; CKD, chronic kidney disease; CVD, cardiovascular disease; FBG, fasting blood glucose; HbA1c, glycated hemoglobin; WC, waist circumference; TG, triglycerides; eGFR, estimated glomerular filtration rate; MACE, major adverse cardiovascular events; LDL-C, low-density lipoprotein cholesterol; RAAS, renin-angiotensin-aldosterone system; BP, blood pressure; DKD, diabetic kidney disease; ADPKD, autosomal dominant polycystic kidney disease.

### 2.2 Bibliometric analysis and main findings

To further characterize the publication trend and research structure of this field, a bibliometric analysis was conducted using records retrieved from the Web of Science Core Collection. Publications published between 2010 and 2025 were exported with full records and cited references. After data screening and preparation, a total of 5,916 publications were included in the bibliometric analysis. VOSviewer version 1.6.20 and Microsoft Excel 2021 were used for visualization and descriptive analysis. To improve the consistency of keyword analysis, a thesaurus file was applied to merge synonymous or highly similar terms, standardize keyword presentation, and remove overly general terms. Three analyses were performed: annual publication trend analysis, country co-authorship analysis, and author keyword co-occurrence analysis.

**Figure 1:**
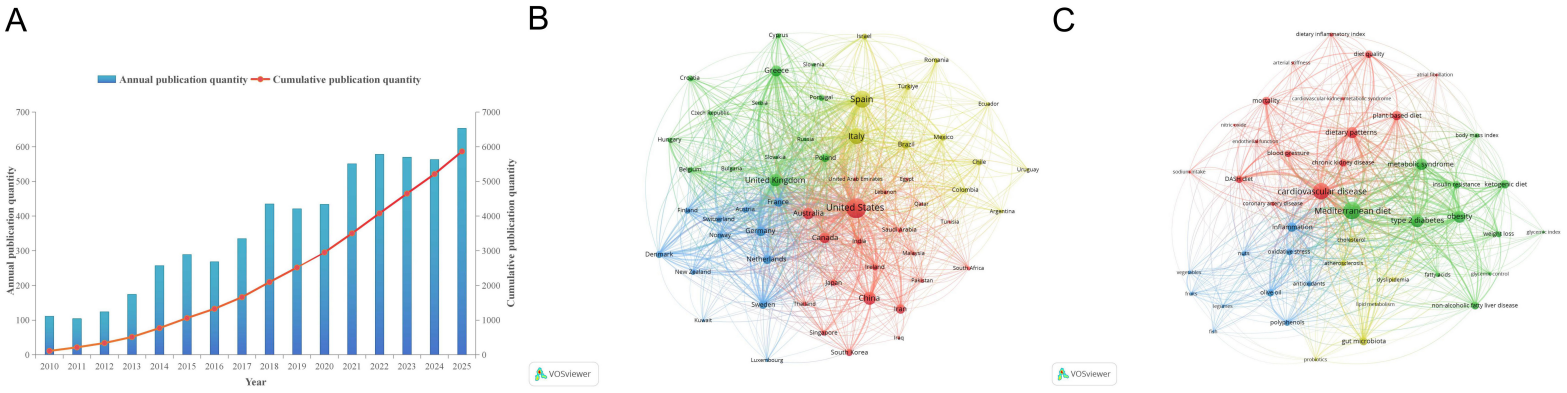
Bibliometric profile of the included publications on dietary patterns and CKM-related conditions from 2010 to 2025. **(A)** Annual and cumulative publication trends of studies related to dietary patterns and CKM-related diseases. **(B)** International collaboration network among countries. **(C)** Author keyword co-occurrence network.

Publications on dietary patterns and CKM-related diseases showed an overall upward trend from 2010 to 2025, with the highest annual output observed in 2025 (Figure 1A). This upward trend suggests that the role of dietary patterns in metabolic, renal, and cardiovascular health has received increasing scholarly attention. Moreover, Supplementary Tables S1–S3 summarize the top 20 authors, journals, and countries contributing to this field.

The international collaboration network showed several distinct clusters of cooperation among countries (Figure 1B). The red cluster mainly included the United States, China, Canada, and Australia; the green cluster included the United Kingdom, Greece, Poland, and Portugal; the blue cluster included Germany, the Netherlands, France, Sweden, and Denmark; and the yellow cluster included Italy, Spain, Brazil, Mexico, and Chile. The strongest collaborative link was observed between the United States and Spain, followed by the United States–Italy and Spain–Italy links, indicating close international collaboration among major contributors in this field.

Through co-occurrence analysis of author keywords, four major clusters were identified in the network map (Figure 1C). The red cluster, including cardiovascular disease, dietary patterns, plant-based diet, DASH diet, mortality, blood pressure, chronic kidney disease, diet quality, and cardiovascular–kidney–metabolic syndrome, mainly reflected the associations between dietary patterns and cardio-renal outcomes. The green cluster, represented by Mediterranean diet, type 2 diabetes, obesity, metabolic syndrome, insulin resistance, ketogenic diet, and glycemic control, focused on metabolic dysfunction and weight- or glucose-related outcomes. The blue cluster, including inflammation, oxidative stress, antioxidants, polyphenols, olive oil, nuts, vegetables, and fruits, highlighted dietary components and potential inflammation- or oxidative stress-related mechanisms. The yellow cluster, comprising gut microbiota, dyslipidemia, atherosclerosis, probiotics, cholesterol, and lipid metabolism, reflected mechanistic themes linking the gut microbiota, lipid metabolism, and atherosclerosis.

Based on the bibliometric findings, structured evidence mapping, and the thematic scope of this review, the subsequent sections are organized as follows. First, we provide an overview of CKM syndrome in relation to major dietary patterns and dietary quality indices. Second, we outline the pathophysiological continuum of CKM syndrome and its core components. Thereafter, we summarize evidence on the associations between major dietary patterns and the core components of CKM syndrome. Potential biological mechanisms, including inflammation, oxidative stress, endothelial dysfunction, gut microbiota, lipid metabolism, and atherosclerosis, are discussed within the corresponding sections.

## 3 Overview of diet and CKM syndrome

### 3.1 Major dietary patterns and CKM risk

Currently, the primary dietary patterns used to evaluate cardiometabolic-renal health include MedDiet, DASH diet, PBD, and KD. Existing evidence suggests the potential regulatory role of specific patterns on overall CKM risk. A large-scale analysis based on NHANES 2011–2020 showed that higher DASH scores were independently associated with lower CKM stages, suggesting a potential association with more favorable cardiometabolic-renal profiles (6). Furthermore, studies focusing on patients with moderate-to-severe CKD suggested that a healthy PBD was associated with a lower burden of CKM risk factors without increasing the risk of hyperkalemia, supporting its metabolic safety and suggesting potential relevance to CKM-related risk reduction and management (7). Although direct evidence linking the MedDiet and KD to CKM syndrome at a holistic level is currently lacking, given their demonstrated benefits on CKM core components (CVD, T2DM, CKD, and MetS), analyzing their impact on individual components remains a key approach to evaluating their value in interrupting CKM progression.

### 3.2 Dietary quality indices and CKM risk

While single dietary patterns are difficult to quantify precisely, structured dietary quality indices provide standardized tools to evaluate potential associations with CKM. These indices help characterize the relationship between diet and CKM progression by quantifying factors such as dietary inflammation, antioxidant capacity, and microbiome modulation. First, indices targeting core CKM features have demonstrated observational associations with disease risk. The dietary inflammatory index (DII) has been frequently associated with advanced CKM risk and all-cause mortality in epidemiological studies (8, 9). Additionally, higher scores on the composite dietary antioxidant index (CDAI) have been linked to a lower prevalence of CKM Stages 3–4 (10). Second, the quality of food sources, rather than mere plant-based attributes, is associated with the direction of impact on CKM. Stratified research based on the PBD index (PDI) found that only the healthy PDI (hPDI) was associated with reduced cardiovascular and all-cause mortality risks in CKM patients, whereas the unhealthy PDI (uPDI) was associated with increased mortality risk (11). These findings highlight the importance of considering the processing level and health attributes of plant foods. Finally, novel indices integrating the microbiome and ecological sustainability have expanded the dimensions of CKM research. The gut microbiome health index (DI-GM), which quantifies dietary modulation of the flora, has been shown to be negatively correlated with CKM risk. This association appears to be partially mediated by phenotypic age acceleration and inflammatory markers, suggesting that gut microbiota-related pathways warrant further investigation as potential mediators of the diet-CKM interaction (12, 13). Additionally, high scores on the planetary health diet index (PHDI)—a tool bridging individual health and environmental sustainability—are associated with a decreased risk of advanced CKM, suggesting that environmentally friendly dietary structures may potentially align with both metabolic gain and ecological protection (14). Overall, as shown in Table 2, dietary quality indices may provide quantitative tools for characterizing dietary exposure in relation to CKM and for generating mechanistic hypotheses for future investigation. Given that existing evidence is mostly derived from cross-sectional data, future research is warranted to investigate potential causal relationships between these indices and CKM stage evolution in longitudinal cohorts.

**Table 2:** Impact of dietary quality indices on CKM syndrome.

Dietary quality index	Core focus/definition	Observed CKM associations	Potential biological relevance
Dietary inflammatory index (DII)	Quantifies the pro-inflammatory potential of the diet	Significant positive association with advanced CKM risk and all-cause mortality (8)	May reflect pro-inflammatory exposure and possible aging-related pathways (9)
Composite dietary antioxidant index (CDAI)	Reflects adequate intake of antioxidants like Vit C/E, Selenium, and Zinc	Closely linked to a reduced prevalence of CKM stages 3–4 (10)	Suggests a possible role of antioxidant-related pathways (10)
Healthy plant-based diet index (hPDI)	Emphasizes the quality and healthy attributes of non-processed plant foods	Associated with lower cardiovascular and all-cause mortality risks in CKM patients (11)	Highlights the importance of plant-food quality and processing level
Unhealthy plant-based diet index (uPDI)	Focuses on high processing levels and unhealthy attributes of plant foods	Associated with higher mortality risk in CKM patients (11)	Suggests that higher processing may be linked to less favorable outcomes
Gut microbiome health index (DI-GM)	Quantifies dietary modulation of the gut flora	Negatively correlated with CKM risk (12, 13)	May be partially related to phenotypic age acceleration and inflammation-related pathways (12, 13)
Planetary health diet index (PHDI)	Bridges individual health with environmental sustainability	High scores are significantly associated with a decreased risk of advanced CKM (14)	Suggests potential alignment between metabolic health and environmental sustainability

CKM, cardiovascular-kidney-metabolic. The DII quantifies the inflammatory potential of the diet based on 45 food parameters linked to biomarkers such as CRP and IL-6 (Ref: PMID: 23941862). Higher scores indicate pro-inflammatory effects, while lower or negative scores reflect anti-inflammatory patterns. The CDAI is a composite score representing the total dietary antioxidant intake based on vitamin A, C, E, selenium, zinc, and carotenoids (Ref: PMID: 15229119). Higher scores reflect a stronger antioxidant potential of the diet. The hPDI and uPDI evaluate the quality of plant-based dietary patterns by categorizing foods into healthy plant foods, unhealthy plant foods, and animal foods. For hPDI, positive scores are assigned to healthy plant foods, while unhealthy plant and animal foods receive reverse scores. For uPDI, positive scores are assigned to unhealthy plant foods, while healthy plant and animal foods receive reverse scores (Ref: PMID: 27299701). The DI-GM represents a literature-based score designed to capture dietary components related to gut microbiota profiles. The calculation involves 14 food groups, including beneficial components such as whole grains and fiber, as well as detrimental components like processed meat. Higher scores indicate a diet more conducive to a healthy gut microbiome (Ref: PMID: 38613077). The PHDI measures adherence to a sustainable health diet based on 14 food groups: whole grains, fruits, vegetables, nuts, legumes, oils, fish, starchy vegetables, dairy, meat, poultry, eggs, fats, and sugars (Ref: PMID: 39985957). Higher scores reflect a dietary pattern supporting both human health and environmental sustainability.

## 4 Pathophysiology continuum of CKM syndrome

### 4.1 Overview of CKM syndrome pathophysiology

CKM syndrome encompasses four core components: MetS, T2DM, CKD, and CVD. Fundamentally, this is not merely a state of multimorbidity, but a pathophysiological continuum characterized by metabolic dysregulation and constituted by multi-organ interactions (3). The disease course initiates with adipose tissue dysfunction (Stage 1), where visceral adipose tissue may initiate systemic inflammation by releasing pro-inflammatory factors and inducing insulin resistance. Subsequently, established MetS and T2DM (Stage 2) act as key drivers, accelerating systemic vascular injury through glucotoxicity and oxidative stress. In this process, CKD exhibits significant bidirectional pathological characteristics: it acts as a downstream target organ of early metabolic injury (Stage 2), while upon progressing to moderate-to-advanced stages (Stage 3), it transforms into a potent amplifier of cardiovascular risk, exacerbating myocardial remodeling through increased volume load and accumulation of uremic toxins. Ultimately, these pathological changes converge upon CVD (Stage 3–4) as the clinical endpoint, completing the evolution from upstream subclinical metabolic risk to downstream structural and functional failure of multiple organs. Recognizing the dynamic evolution of this continuum, the following sections discuss the potential impact of specific dietary patterns on the progression of CKM syndrome, covering the spectrum from early metabolic risk to target organ injury.

### 4.2 mTOR signaling pathway in CKM progression

The mechanistic target of rapamycin (mTOR) signaling pathway functions as a conserved regulator of nutrient sensing, and its dysregulation has been increasingly associated with the pathophysiological continuum of CKM syndrome. Experimental studies suggest that pathway imbalances may correlate with the transition from early metabolic issues to organ failure (15–17). During the Stage 1–2, preclinical research indicates chronic nutrient overload might activate Rab1A–mTORC1 in β cells, affecting the transcription factor PDX1 (18, 19). Specifically, in cell models, mTORC1 has been observed to phosphorylate PDX1 at Ser61 (20). While this mechanism potentially relates to obesity and hepatic steatosis, its causal role in humans remains to be validated. As the disease reaches Stage 3–4, preliminary data suggests nuclear mTOR signaling may participate in the link between metabolic stress and cellular senescence (21). Laboratory findings associate these pathways with kidney injury and atherosclerosis (22–24). Additionally, muscle models suggest chronic mTORC1 activation might correlate with mitochondrial dysfunction and aging (25–27). Overall, these mTOR-related mechanisms offer a biological perspective for CKM transitions.

### 4.3 Sex and gender in CKM progression

Sex- and gender-related factors represent an important yet underexplored dimension of the CKM syndrome and may influence both disease progression and responses to dietary interventions (28, 29). Biological sex differences in blood pressure trajectories, hormonal milieu, and organ vulnerability may contribute to heterogeneity in CKM risk across the life course (28, 30). In parallel, gender-related determinants, including dietary behaviors, healthcare access, and treatment adherence, may further shape diet quality and CKM outcomes (29, 31, 32). Female-specific hormonal transitions, particularly after menopause, may add further complexity to cardiometabolic risk profiling and the interpretation of dietary effects in women (33). Available evidence also suggests sex-specific heterogeneity in responses to dietary patterns, although the current data remain limited and sometimes inconsistent (34–36). Therefore, future studies should incorporate sex-stratified analyses and gender-informed variables to better understand heterogeneity in dietary associations across the CKM syndrome.

## 5 MedDiet

**Figure 2:**
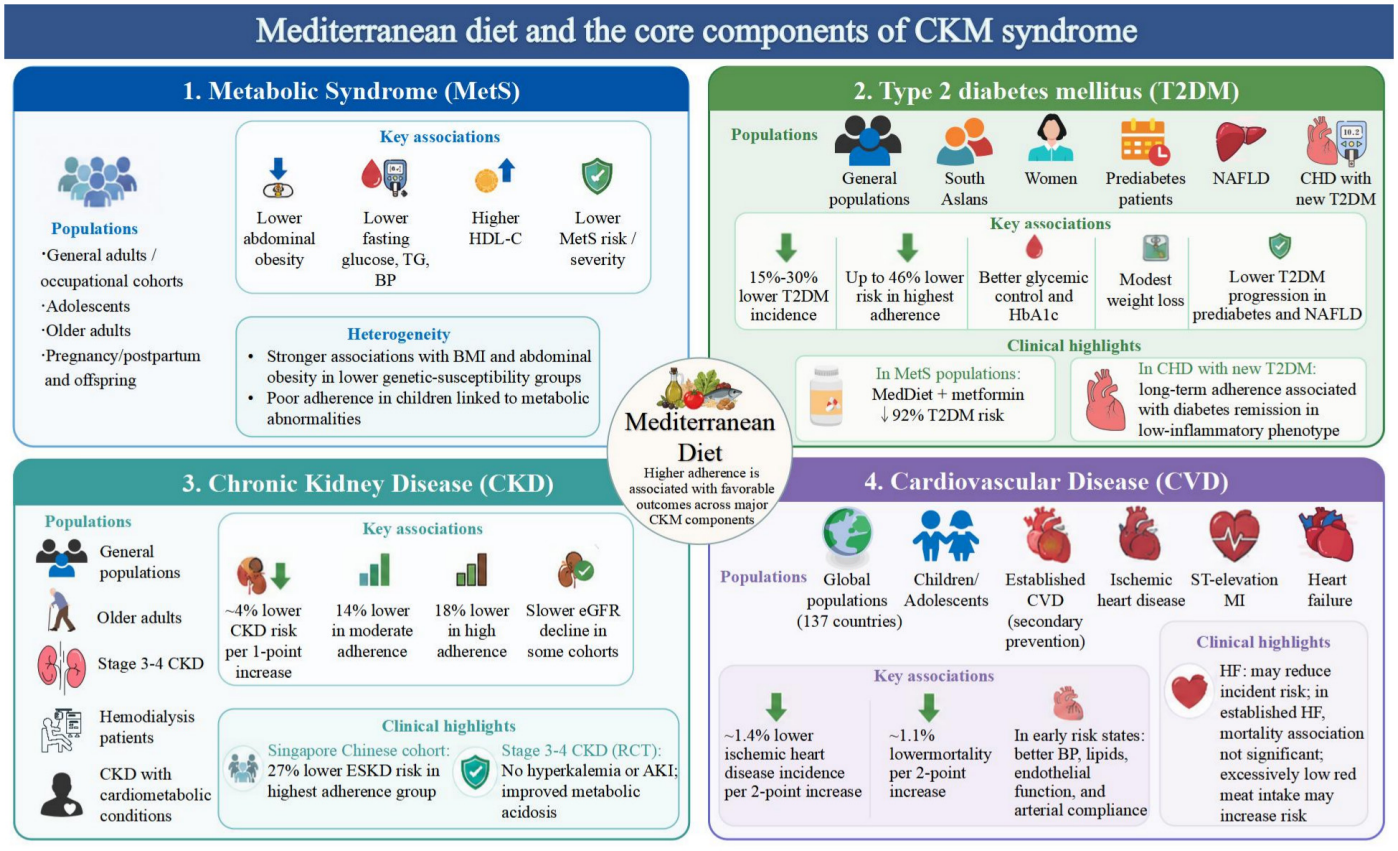
Mediterranean diet and the core components of CKM syndrome. MedDiet may support favorable CKM-related outcomes through anti-inflammatory, antioxidant, metabolic, vascular, and gut microbiota–related pathways. These mechanisms may contribute to lower MetS, T2DM, CKD, and CVD risks, although associations vary across populations and disease stages. CKM, cardiovascular-kidney-metabolic; MedDiet, Mediterranean diet; MetS, metabolic syndrome; T2DM, type 2 diabetes mellitus; CKD, chronic kidney disease; CVD, cardiovascular disease; BMI, body mass index; TG, triglycerides; BP, blood pressure; HDL-C, high-density lipoprotein cholesterol; HbA1c, glycated hemoglobin; NAFLD, non-alcoholic fatty liver disease; CHD, coronary heart disease; eGFR, estimated glomerular filtration rate; ESKD, end-stage kidney disease; AKI, acute kidney injury; HF, heart failure; MI, myocardial infarction.

The MedDiet reflects the traditional dietary habits of Mediterranean populations and is characterized by a high intake of plant-based foods and olive oil, moderate consumption of fish and dairy products, and limited intake of red and processed meats as well as refined sugars (37). Figure 2 summarizes the associations between the MedDiet and the core components of CKM syndrome.

### 5.1 MedDiet and MetS

#### 5.1.1 Epidemiological associations

Epidemiological and clinical evidence generally supports the MedDiet as an effective strategy for the prevention and management of MetS. Cross-sectional analyses from NHANES (2007–2020) and large occupational cohorts have shown an inverse, dose-dependent association between MedDiet adherence and MetS prevalence (38, 39), with similar findings reported in US career firefighters and Spanish university students (40, 41). Prospective and interventional studies further support a beneficial role of Mediterranean dietary patterns in MetS. In particular, greater adherence has been associated with lower MetS risk, higher reversal rates, and lower MetS severity scores, especially when combined with energy restriction or physical activity (42–44). In addition, long-term adherence to the MedDiet has been linked to lower all-cause and cardiovascular mortality in patients with MetS (45, 46).

Nevertheless, findings are not entirely consistent across populations. Studies conducted in non-Mediterranean settings, including US adolescents, high-risk cardiovascular populations in New Zealand, and older adults, did not observe significant associations between the MedDiet and MetS prevalence (47–49). This heterogeneity may reflect differences in dietary adherence and host susceptibility. For example, poor adherence in Greek children was associated with higher risks of central obesity and hypertriglyceridemia (50), while the HELENA study suggested that the beneficial associations of the MedDiet with body mass index (BMI) and abdominal obesity were more evident in individuals with lower genetic susceptibility to obesity (51). Together, these findings indicate that although the MedDiet shows overall benefit for MetS, its effects may vary according to population characteristics, adherence, and genetic background.

#### 5.1.2 Relationship with core components and special populations

The improvement of metabolic health by the MedDiet demonstrates benefits across multiple components of MetS, and these benefits often precede the complete reversal of the clinical syndrome diagnosis. Multiple studies indicate that the MedDiet is associated with positive changes in all core parameters, including waist circumference (WC), blood pressure, fasting blood glucose, and lipid metabolism markers [riglycerides (TG), high-density lipoprotein (HDL)] (52, 53). Interventions targeting specific adolescent females with MetS further supported these observations: MedDiet intervention significantly improved body weight, blood lipids, and fasting blood glucose, while reducing key inflammatory markers (interleukin-6 [IL-6], high-sensitivity C-reactive protein [hs-CRP]), suggesting its potential dual role in ameliorating metabolic disorders and suppressing inflammation (54). Of particular note is its effect on body composition: in elderly MetS populations, the MedDiet is associated with reduced visceral fat while preserving skeletal muscle mass (55). Notably, the metabolic protective effects of the MedDiet may extend across the life course. Dietary intervention during pregnancy and postpartum not only significantly reduces maternal postpartum risk of MetS and glucose abnormalities (56)but is also associated with lower oxidative stress levels and MetS risk in offspring during adolescence, suggesting its benefits may influence the metabolic programming of the next generation via the intrauterine environment (57).

#### 5.1.3 Potential biological mechanisms

The MedDiet ameliorates MetS through a multi-dimensional mechanism involving molecular signaling, structural remodeling, and metabolic recalibration. At the molecular level, the diet provides specific ligands to modulate inflammation beyond simple NF-κB downregulation (58). Specifically, MedDiet polyphenols and ω-3 fatty acids act as PPAR-γ and GPR120 agonists, antagonizing TLR4 signaling in adipocytes and macrophages (59, 60). This targeted blockade reduces pro-inflammatory cytokines, including IL-1β, IL-6, and TNF-α (58). Simultaneously, the MedDiet is associated with reduced oxidative stress, characterized by lower myeloperoxidase (MPO) activity and malondialdehyde (MDA) levels, particularly when combined with physical activity (61). These changes coincide with improvements in blood glucose, blood pressure, and lipid profiles (61). Additionally, recent omics data show the MedDiet induces a functional shift in host-microbiome co-metabolism (62). High fiber intake enriches SCFA-producing taxa like Faecalibacterium prausnitzii, elevating systemic butyrate levels (63), thereby improving insulin sensitivity (64).

### 5.2 MedDiet and T2DM

#### 5.2.1 Incidence risk and primary prevention

Large-scale epidemiological evidence consistently supports the potential role of the MedDiet in the primary prevention of T2DM. Multiple prospective cohort studies and analyses from randomized and observational studies show that higher MedDiet adherence is significantly inversely associated with the risk of developing T2DM, with risk reductions typically ranging from 15% to 30% (65–67), and reaching up to 46% in the highest adherence groups (68). The Mediterranean lifestyle (MEDLIFE), which integrates diet, physical activity, rest, and social behavior, has also been associated with an approximate 30% reduction in T2DM risk (69). Furthermore, the observed benefits of this dietary pattern appear to be consistent across diverse ethnic and physiological boundaries. In South Asian and female cohorts, high adherence was associated with a significantly reduced diabetes risk, potentially through the improvement of insulin resistance and lipoprotein metabolism (70, 71). For specific high-risk populations, the MedDiet also demonstrates considerable preventive potential, significantly reducing the risk of progression to T2DM in individuals with prediabetes (72, 73) and non-alcoholic fatty liver disease (74, 75). However, findings from a non-Mediterranean region of Switzerland were not significant, suggesting that cultural adaptability may influence the generalizability of this dietary pattern (76).

#### 5.2.2 Disease remission and treatment synergy

For patients with established T2DM or prediabetes, RCTs provide robust evidence supporting the role of the MedDiet in improving glucose homeostasis. Overall, MedDiet intervention is associated with better glycemic control, lower hemoglobin A1c (HbA1c) levels, and modest weight loss compared with various control diets (77–81). Importantly, these benefits may not depend entirely on weight reduction, as an isocaloric restriction trial in a Chinese prediabetic population showed that even in a weight-stable state, the MedDiet improved insulin resistance more significantly than a traditional diet, indicating potential metabolic regulatory effects of the dietary structure itself (82).

The MedDiet may also complement pharmacotherapy. In populations with MetS, the combination of MedDiet and metformin reduced the risk of T2DM by 92%, significantly outperforming drug therapy alone (83). In addition, the CORDIOPREV study showed that in coronary heart disease patients with newly diagnosed T2DM, long-term adherence to the MedDiet helped contribute to diabetes remission in those with a low-inflammatory phenotype (84). This observation supports the possibility that host inflammatory status may influence the metabolic response to dietary intervention. Additionally, metabolomic profiling has revealed that the MedDiet can ameliorate dysregulation in metabolites associated with glycolysis and the tricarboxylic acid cycle, which are typically linked to T2DM risk, providing further mechanistic support for its metabolic benefits (85).

#### 5.2.3 Potential biological mechanisms

The mechanisms by which the MedDiet improves glucose metabolism are multifaceted. First, the restoration of insulin sensitivity is the central pivot. Epidemiological and interventional studies suggest that the MedDiet rapidly improves beta-cell function and insulin clearance, an effect often superior to low-carbohydrate diets alone (86). Second, anti-inflammatory and antioxidant effects may help mitigate metabolic stress. The abundance of polyphenols and flavonoids in the diet (e.g., citrus flavonoids) is associated with lower levels of pro-inflammatory cytokines like IL-6 (87), while a lower intake of advanced glycation end-products (AGEs) correlates with reduced oxidative injury (88). Finally, the gut microbiota is proposed as a potential mediator. Rich dietary fibers promote the proliferation of short-chain fatty acid-producing bacteria; these metabolites not only maintain the intestinal barrier but also directly enhance insulin sensitivity (89). Interestingly, this relationship appears bidirectional: the MedDiet was associated with increased microbial richness and shifts in community composition in T2DM patients, changes which were found to correlate with improvements in HbA1c and insulin resistance (90), while higher baseline microbial diversity was associated with greater metabolic benefits from the diet (91).

### 5.3 MedDiet and CKD

#### 5.3.1 Incidence risk and primary prevention

Evidence generally suggests that higher adherence to the MedDiet is associated with a lower risk of incident CKD and end-stage kidney disease (ESKD). A cross-sectional study involving 33,015 participants showed that for every 1-point increase in MedDiet adherence, the risk of CKD decreased by approximately 4%, with risks in the moderate and high adherence groups reduced by about 14% and 18%, respectively (92). A large-scale cohort study based on the UK Biobank (n = 207,268) also observed that compared to those with the lowest adherence, individuals with high adherence had a significantly lower risk of developing CKD (93), an association that was broadly consistent across other studies (94, 95). This association exhibits a dose-dependent pattern; data from the Singapore Chinese Health Study showed that as alternate MedDiet score (aMED) increased, the risk of ESKD declined in a tiered manner, with a 27% reduction in the highest quintile (96). These associations have also been observed across different age groups. In the elderly, high adherence is associated with a significant slowing of natural renal function decline (97); while in children, a good MedDiet pattern was found to significantly reduce the risk of transient glomerular injury, suggesting a potential protective role of early dietary intervention on glomerular function (98). However, findings are not entirely consistent. NHANES data shows that high MedDiet adherence combined with regular physical activity is linked to the maximum benefit for reducing CKD risk (92). Although the vast majority of evidence supports the protective role of the MedDiet, some cross-sectional studies did not find a statistically significant association (99), which may be related to racial differences, heterogeneity of dietary assessment tools, or limitations of the cross-sectional design.

#### 5.3.2 Disease progression and prognosis improvement

For high-risk patients with established CKD or comorbid cardiometabolic conditions, the MedDiet demonstrates dual potential in delaying disease progression and improving survival prognosis. Long-term follow-up data indicates that high adherence not only significantly reduces the risk of CKD progression but also substantially decreases all-cause mortality events (100). Interventional studies further supported these benefits in specific high-risk phenotypes: in obese elderly populations with MetS, an energy-restricted MedDiet effectively slowed the rate of estimated glomerular filtration rate (eGFR) decline (101, 102); while in coronary heart disease patients with Type 2 Diabetes, a MedDiet rich in extra virgin olive oil was superior to a traditional low-fat diet in mitigating renal function deterioration and proteinuria progression (103, 104). Even upon progression to ESKD, this pattern remains positively correlated with better nutritional reserves (protein and omega-3 fatty acid intake) and physical performance in hemodialysis patients (105). Crucially, clinical concerns regarding the safety of high-potassium diets were addressed by a randomized crossover trial in Stage 3–4 CKD patients: this dietary pattern did not precipitate hyperkalemia or acute kidney injury, but rather showed improvements over conventional diets in metabolic acidosis (106), suggesting that under close monitoring, the MedDiet is a safe and metabolically advantageous strategy for CKD management.

#### 5.3.3 Potential biological mechanisms

The potential renal protective effects of the MedDiet may be mediated by several interrelated pathways, including antioxidant activities, metabolic regulation, and gut microbiota modulation. First, extra virgin olive oil—a core component—contains bioactive substances such as polyphenols that are suggested to modulate oxidative stress and vascular endothelial function, which may correlate with reduced renal inflammatory burden and proteinuria (107). Second, metabolic profiles associated with the MedDiet appear to play a significant role. Prospective cohort evidence indicates that specific serum metabolites linked to fish intake are associated with a reduced risk of incident CKD (108). Finally, the gut-kidney axis represents a postulated pathway, with observational data linking higher MedDiet adherence to lower circulating levels of the microbiota-derived uremic toxin p-cresyl sulfate (109).

### 5.4 MedDiet and CVD

#### 5.4.1 Incidence risk and primary prevention

The MedDiet currently possesses extensive evidence for cardiovascular protection and is widely regarded as a key strategy to impede the transition of CKM syndrome from early metabolic risk to clinical CVD. At the macro-epidemiological level, an ecological study covering 137 countries with a 27-year follow-up showed that for every 2-point increase in MedDiet score, the incidence and mortality of ischemic heart disease decreased by approximately 1.4% and 1.1%, respectively, supporting its global benefits for cardiovascular health (110). Multiple cohort studies further indicated that this pattern was associated with a lower risk of CVD (111–113), including coronary heart disease (114, 115), atrial fibrillation (116), hypertension (117), and subclinical atherosclerosis (118). During CKM Stages 1–2, the MedDiet exhibits strong regulatory capabilities. For high-risk groups with obesity, pre-hypertension, or metabolic abnormalities, improving adherence not only is associated with optimized blood pressure (119, 120)and lipid profiles (121, 122) but, critically, has been linked to improved endothelial dysfunction and arterial compliance (123–125). This early repair of the vascular microenvironment, accompanied by reduced carotid plaque burden (126) and visceral fat reduction (127), may help mitigate the pathological drivers of cardiovascular events. Furthermore, in children and adolescents, high adherence is associated with better cardiometabolic phenotypes (128, 129), highlighting the potential relevance of early-life diet to CKM health.

#### 5.4.2 Disease management and secondary prevention

For patients with established CVD, the value of the MedDiet may involve reducing residual risk and generating synergistic effects with pharmacotherapy. In the secondary prevention of coronary heart disease, the MedDiet shows benefits superior to traditional low-fat diets (130). The CORDIOPREV study suggested that long-term adherence to the MedDiet was associated with slower arterial plaque progression and a lower risk of major adverse cardiovascular events (131). Similar findings have been reported in patients with prior ischemic heart disease (132). Particularly in the acute phase management of ST-segment elevation myocardial infarction, high adherence is associated with better inflammation control (e.g., rapid decline of hs-CRP) and reperfusion outcomes (133), highlighting the potential of the diet in the management of post-acute coronary syndrome. However, in the terminal stage of heart failure (HF), dietary intervention presents complexity. Although meta-analyses suggest the MedDiet may reduce the risk of developing HF (134), NHANES data reveals that in patients with established HF, the association between total MedDiet score and mortality risk was not statistically significant; conversely, excessively low red meat intake might be associated with increased mortality risk (135). These findings suggest that the potential benefits of the MedDiet may differ between HF prevention and established HF, and that its role in patients with advanced disease remains uncertain. Additionally, gender differences warrant attention, as some studies suggest the preventive effect on heart failure may be weaker in women (36), although the evidence remains limited. Overall, current evidence supports a possible role for the MedDiet in secondary prevention, but heterogeneity across outcomes and populations should be acknowledged.

#### 5.4.3 Potential biological mechanisms

The MedDiet appears to modulate the shared pathological environment of CVD through multi-targeted mechanisms, centering on systemic vascular endothelial protection, metabolism reprogramming, and inflammatory modulation. First, endothelial dysfunction represents a key early stage of cardiovascular injury. Randomized trials suggest that the MedDiet correlates with increased circulating endothelial progenitor cells and reduced endothelial microparticles (136). Bioactive components, particularly olive oil phenols, are suggested to support nitric oxide bioavailability, which may correlate with improved vascular tone and arterial stiffness (137). Second, the MedDiet not only modulates lipid composition through increased omega-3 intake (138) but also may influence the expression of cholesterol efflux transporters (ABCA1/ABCG1), potentially supporting reverse cholesterol transport (139). Simultaneously, the MedDiet is associated with favorable shifts in atherothrombotic biomarkers in high-risk populations, suggesting a potential role in modulating the risk of acute cardiovascular events (140). Finally, regarding the inflammatory background of CVD, systematic reviews suggest the MedDiet may modulate Nrf2/ARE and NF-κB signaling, which is associated with lower levels of IL-6, TNF-α, and hs-CRP (141). However, the direct impact of these mechanistic pathways on clinical outcomes, such as plaque stabilization, requires further validation through robust clinical trials.

## 6 DASH diet

**Figure 3:**
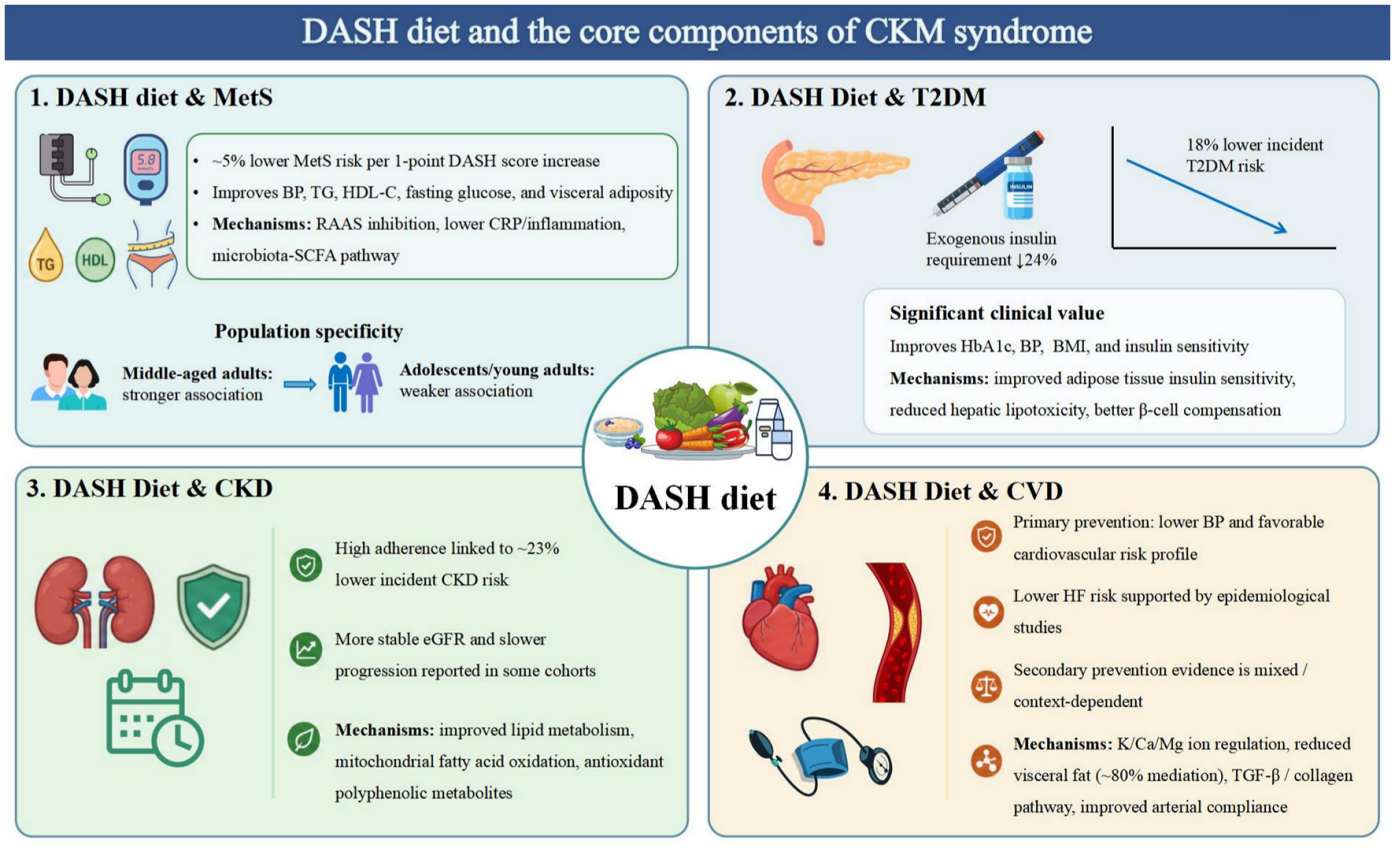
DASH diet and the core components of CKM syndrome. DASH is associated with favorable CKM-related outcomes through blood pressure control, improved glucose–lipid metabolism, reduced inflammation, microbiota–SCFA modulation, antioxidant effects, and vascular protection, although responses may differ across populations and disease stages. DASH, Dietary Approaches to Stop Hypertension; CKM, cardiovascular-kidney-metabolic; MetS, metabolic syndrome; T2DM, type 2 diabetes mellitus; CKD, chronic kidney disease; CVD, cardiovascular disease; BP, blood pressure; TG, triglycerides; HDL-C, high-density lipoprotein cholesterol; CRP, C-reactive protein; RAAS, renin-angiotensin-aldosterone system; SCFA, short-chain fatty acids; HbA1c, glycated hemoglobin; BMI, body mass index; eGFR, estimated glomerular filtration rate; HF, heart failure; K, potassium; Ca, calcium; Mg, magnesium; TGF-β, transforming growth factor-β.

The DASH diet, originally proposed by the US National Institutes of Health, emphasizes vegetables, fruits, whole grains, low-fat dairy, nuts, and plant proteins, while limiting sodium, added sugars, saturated fats, and processed meats (142). As illustrated in Figure 3, the DASH diet is associated with favorable effects across the major components of CKM syndrome.

### 6.1 DASH diet and MetS

#### 6.1.1 Epidemiology and clinical intervention

Substantial cross-sectional and longitudinal research indicates that adherence to the DASH diet is significantly inversely associated with the risk of developing MetS. Cross-sectional studies in US adults and a large-scale Iranian population generally support this inverse association (34, 143), and Konikowska et al. further quantified this association, noting that for every 1-point increase in DASH diet quality score, the risk of MetS decreased by 5% (OR = 0.95) (144). However, this protective effect exhibits heterogeneity across age groups and cultural backgrounds. The NDNS longitudinal study found that this association was mainly evident in middle-aged populations, while the association weakens in adolescents and young adults (145), consistent with findings from another case-control study in adolescents (146). Furthermore, a study of suburban residents in Shanghai found no significant association (147), suggesting that the applicability of DASH-based assessment may vary across populations. A systematic review and meta-analysis of intervention studies suggested that the DASH diet has significant advantages over other dietary or lifestyle interventions in improving cardiovascular and metabolic risk factors in MetS patients (148). A network meta-analysis of multiple RCTs further indicated that DASH performed favorably among common dietary patterns for MetS management (149). Moreover, multimodal strategies incorporating the DASH diet together with aerobic exercise or behavioral interventions may offer greater benefits than single-component interventions (150).

#### 6.1.2 Effects on core components

The ameliorating effect of the DASH diet on MetS is concentrated on the multidimensional regulation of its core components (blood pressure, lipids, glucose, and central obesity). As the most prominent feature of this dietary pattern, blood pressure control is consistently supported by meta-analyses, where intervention significantly reduced systolic and diastolic blood pressure (148), and the antihypertensive effect in MetS patients was superior to other common dietary strategies (149). In terms of lipid metabolism, studies consistently show that DASH diet scores are inversely correlated with TG and positively correlated with high-density lipoprotein cholesterol (HDL-C) (143, 144). DASH adherence has also been associated with lower fasting blood glucose (143, 151, 152). Notably, recent research has begun to focus on novel composite metabolic indices. RCTs demonstrate that DASH diet intervention significantly reduces the triglyceride-glucose (TyG) index and lipid accumulation product (152), suggesting a broader effect on glucolipid dysregulation. Additionally, abdominal obesity is a core driver of MetS. Multiple studies indicate that the DASH diet is associated with reductions in body weight and significantly decreases WC and visceral fat deposition (144, 153), supporting its overall metabolic benefits in MetS.

#### 6.1.3 Potential biological mechanisms

The metabolic benefits observed with the DASH diet are likely underpinned by a complex interplay between neurohumoral regulation, oxidative stress, and the immune microenvironment. First, the dietary pattern of DASH, which is characterized by low sodium intake alongside high levels of potassium and magnesium, regulates vascular tone by promoting natriuresis and calcium antagonism, and by inducing the hyperpolarization of vascular smooth muscle. Furthermore, this pattern inhibits the activity of the RAAS to mitigate vasoconstriction and fibrosis (154). Second, immunomodulatory and anti-inflammatory properties are considered potential core mechanisms. Research indicates that high DASH adherence is associated with lower levels of systemic inflammatory markers such as CRP (155). Further cytological studies reveal that the DASH diet correlates with shifts in peripheral leukocyte and lymphocyte subset levels in overweight populations, representing a possible modulation of the immune-inflammatory state at the cellular level that may help alleviate the chronic low-grade inflammatory burden associated with MetS (156). Finally, the DASH diet is proposed to influence the host-microbiome co-metabolism. High fiber intake is linked to the enrichment of SCFA-producing taxa, which may contribute to the regulation of blood pressure and glucose metabolism via GPR41/43 signaling (157–159).

### 6.2 DASH diet and T2DM

#### 6.2.1 Incidence risk and clinical benefits

The DASH diet demonstrates significant clinical value throughout the management of diabetes. In terms of prevention, large-scale meta-analyses suggest that high adherence is associated with an 18% reduction in T2DM incidence risk, exhibiting a linear dose-response relationship (160). For diagnosed patients, this pattern not only improves HbA1c, blood pressure, and BMI (161) but also demonstrates a significant “insulin-sparing effect” in the short term, reducing exogenous insulin requirements by 24% after just 1 week of intervention, suggesting rapid reversal of insulin resistance (162). In addition to these short-term benefits, higher DASH adherence has also been associated with lower all-cause mortality risk (163). However, in high-risk diabetic populations, combined adherence to the DASH diet and the MedDiet may be associated with greater survival benefit than either pattern alone (164). Notably, the DASH diet did not increase daily food costs in insulin-treated patients with T2DM, supporting its potential practicality as a nutritional strategy (165).

#### 6.2.2 Potential biological mechanisms

Multidimensional pathophysiological mechanisms reveal deep pathways by which the DASH diet improves metabolic homeostasis. Evidence from the German diabetes study highlights a crucial mediation pathway: approximately 47% of the inverse association between DASH scores and hepatic lipid content in T2DM patients is mediated by the amelioration of adipose tissue insulin resistance. This physiological improvement restricts the lipolytic release of free fatty acids, thereby mitigating hepatic lipotoxicity (166). Plant-derived components of the DASH diet may also contribute to improved insulin sensitivity, while the elevation of the disposition index suggests enhanced beta-cell compensatory function (167). Metabolomics and proteomics studies have found that the DASH diet may directly intervene in the molecular pathways of diabetes pathogenesis by regulating apolipoprotein A1, the omega-6/omega-3 ratio, and concentrations of various inflammation and thrombosis-related proteins (168, 169). Although systematic reviews suggest that DASH intervention significantly lowers CRP levels (170), findings for other inflammatory markers remain inconsistent (171), suggesting that its anti-inflammatory effects may vary across populations.

### 6.3 DASH diet and CKD

#### 6.3.1 Incidence risk and primary prevention

Large-scale population cohort studies generally support the protective role of the DASH diet in the primary prevention of CKD. Analysis based on NHANES 2005–2020 data shows that DASH is significantly inversely associated with CKD risk (172). This conclusion appears robust across diverse populations: a prospective study from the UK Biobank found that higher DASH adherence was associated with a 23% reduction in incident CKD risk (95); similarly, findings from the Singapore Chinese Health Study indicated a strong correlation between high adherence and reduced risk of ESKD (96). However, findings are not entirely consistent across populations. For instance, a 6-year follow-up of the HCHS/SOL cohort (predominantly Hispanic/Latino) found no significant association between DASH scores and longitudinal changes in eGFR or urinary albumin-to-creatinine ratio (173). Taken together, current evidence suggests that higher DASH adherence is generally associated with lower CKD risk, although the strength and consistency of this association may vary across ethnic and cultural settings.

#### 6.3.2 Disease progression and prognosis improvement

Studies on patients with established CKD suggest that the DASH diet may improve clinical prognosis, though the weight of evidence varies across outcomes and follow-up duration. The Chronic Renal Insufficiency Cohort (CRIC) study observed that adherence to the DASH diet significantly reduced the risk of CKD progression and all-cause mortality (100). The German CKD study found that higher DASH adherence was associated with preserved eGFR (174), while a meta-analysis further suggested that higher DASH adherence was linked to more stable eGFR during follow-up (175). Nevertheless, some findings suggest limitations of this dietary pattern in specific contexts. In the PREDIMED-Plus study, DASH adherence was unrelated to eGFR changes within 1 year, suggesting that short-term dietary intervention may be insufficient to significantly reverse the decline in filtration rate (102). Evidence for mortality outcomes is also inconsistent, while CRIC results were positive, an analysis based on NHANES (2007–2016) did not find a significant association between DASH scores and all-cause mortality in CKD patients (176). Furthermore, caution is needed when interpreting intervention effects due to early hemodynamic effects. An intervention trial found that a low-sodium DASH diet was associated with a significant decrease in eGFR within 4 weeks (177). This early functional decline may reflect renal hemodynamic regulation following blood pressure reduction (i.e., functional decline) rather than substantive renal injury. Therefore, evaluating the impact of the DASH diet on CKD progression requires comprehensive interpretation combined with long-term follow-up data.

#### 6.3.3 Potential biological mechanisms

Metabolomics provides a robust molecular basis for the renoprotective effects of the DASH diet. Regarding lipid metabolism, an analysis of the CRIC cohort revealed that patients with higher DASH adherence exhibited elevated plasma levels of long-chain unsaturated lipids and unsaturated triacylglycerols, whereas levels of saturated lipids and red-meat-associated plasmalogens were significantly lower (178). This suggests that the DASH diet may protect renal tubular epithelial cells from injury by improving mitochondrial fatty acid oxidation and alleviating lipotoxicity. In terms of antioxidant and anti-inflammatory effects, randomized controlled feeding trials indicated that the DASH diet is associated with increased levels of polyphenolic acids and their gut microbial metabolites in urine (179). These metabolites exhibit potent antioxidant activity, scavenging reactive oxygen species (ROS), inhibiting vascular smooth muscle cell proliferation, and enhancing nitric oxide (NO) bioavailability (179), which may contribute to the improvement of renal function.

### 6.4 DASH diet and CVD

#### 6.4.1 Incidence risk and primary prevention

As a cornerstone of primary prevention for cardiovascular disease, the benefits of the DASH diet are most consistently reflected in blood pressure reduction, as supported by large-scale meta-analyses (142, 180). Moreover, the antihypertensive effect of the DASH diet exhibits synergistic potentiation with other lifestyle interventions. When combined with salt restriction or time-restricted eating strategies, the antihypertensive effect is more pronounced (181, 182). If combined with high-intensity exercise like “stair climbing,” it can further reduce the risk of major adverse cardiovascular events (183). Importantly, this benefit may extend across age groups. For adolescents, it not only lowers peripheral blood pressure but also is associated with lower pulse wave velocity during the subclinical stage of arteriosclerotic change (184). Additionally, the value of the DASH diet in reducing the risk of HF is supported by epidemiological evidence (185). However, the generalizability of the DASH diet remains a subject of debate due to population-specific responses. For instance, prospective evidence failed to find a significant cardiovascular benefit of the DASH diet in diabetic cohorts (186). Similarly, in Chinese children a localized modified dietary pattern showed better predictive value for hypertension risk than the standard DASH score (187). These findings underscore the necessity of developing region-specific dietary guidelines.

#### 6.4.2 Disease management and secondary prevention

Unlike the solid evidence chain in primary prevention, the value of the DASH diet in secondary prevention of CVD faces more complex challenges. Although higher overall diet quality scores have generally been associated with better survival among patients with CVD (188, 189), findings specific to DASH are mixed. No significant associations were observed in the Fasa PERSIAN cohort (190) or in NHANES (191), suggesting that the benefits of DASH in secondary prevention may be limited or context-dependent. In addition, a prospective cohort of myocardial infarction patients receiving contemporary standard therapies, including statins and antiplatelet agents, found that greater DASH adherence was not associated with additional survival benefit (192). This suggests that with the enhanced efficacy of modern secondary prevention drugs, the marginal benefit of lifestyle intervention alone is diminishing. Additionally, heterogeneity in geographic and socioeconomic dimensions further complicates this picture. The absence of DASH benefits in Middle Eastern cohorts (193) and the null results in low-income groups in the Southern Community Cohort Study cohort (194) highlight the limitations of standard DASH scoring systems, which may not adequately capture population-specific dietary characteristics or broader socioeconomic constraints.

#### 6.4.3 Potential biological mechanisms

Modern multi-omics studies have outlined potential mechanisms linking the DASH diet to cardiovascular benefit. At the macroscopic level, NHANES analysis suggested that improvement in central obesity may represent an important mediating pathway, with the waist-to-height ratio mediating up to ~80% of the association, indicating that visceral fat reduction may be closely related to hemodynamic improvement (195). At the ion regulation level, the synergy of potassium, calcium, and magnesium may contribute to blood pressure control by promoting natriuresis and regulating vascular tone (196, 197). At the molecular level, the accumulation of plant-derived metabolites, such as stachydrine and tryptophan betaine (198), together with changes in the TGF-β pathway and collagen-related proteins such as CTHRC1 and PCOLCE (199, 200), has been associated with improved arterial compliance. These effects may also be time-dependent, as reversal of subclinical injury appeared to require at least 12 weeks of intervention (201). Moreover, the sustained suppression of thrombo-inflammatory markers after weight regain suggests the possibility of longer-lasting biological effects (202). Furthermore, gene-diet interactions may help explain interindividual variation in response to the DASH diet (203).

## 7 PBD

**Figure 4:**
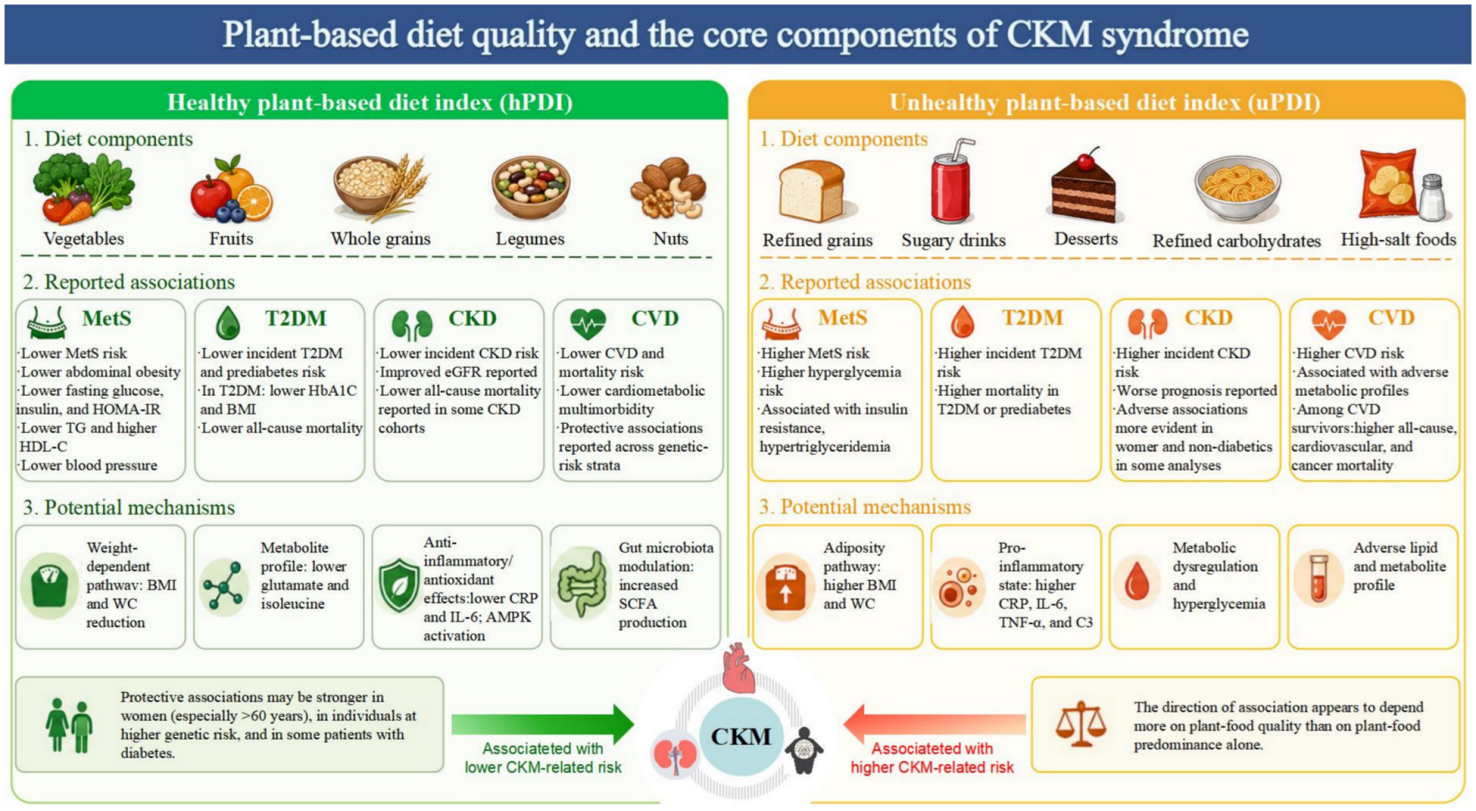
Plant-based diet quality and the core components of CKM syndrome. The hPDI may be associated with lower CKM-related risk through weight control, improved metabolite profiles, anti-inflammatory and antioxidant effects, AMPK activation, and gut microbiota–SCFA modulation. In contrast, uPDI may be associated with higher CKM-related risk through adiposity, insulin resistance, hyperglycemia, inflammation, oxidative stress, and adverse lipid/metabolite profiles. CKM, cardiovascular-kidney-metabolic; hPDI, healthy plant-based diet index; uPDI, unhealthy plant-based diet index; MetS, metabolic syndrome; T2DM, type 2 diabetes mellitus; CKD, chronic kidney disease; CVD, cardiovascular disease; BMI, body mass index; WC, waist circumference; HbA1c, glycated hemoglobin; HOMA-IR, homeostasis model assessment of insulin resistance; TG, triglycerides; HDL-C, high-density lipoprotein cholesterol; eGFR, estimated glomerular filtration rate; CRP, C-reactive protein; IL-6, interleukin-6; AMPK, AMP-activated protein kinase; SCFA, short-chain fatty acids; TNF-α, tumor necrosis factor-α; C3, complement component 3.

The PBD pattern emphasizes increased intake of plant-source foods such as vegetables, fruits, whole grains, legumes, and nuts, while limiting red meat, processed meats, and high-fat animal products (204). Their clinical associations vary markedly by diet quality, with Figure 4 illustrating the contrasting effects of hPDI and uPDI on CKM outcomes.

### 7.1 PBD and MetS

#### 7.1.1 Epidemiological associations

The association between PBD patterns and MetS is highly dependent on the quality of plant foods. Systematic reviews and meta-analyses show that the hPDI is inversely associated with MetS risk, whereas the uPDI is associated with higher risk (205). Observational evidence from NHANES, the China Health and Nutrition Survey, and a French multicenter study consistently supports a protective association of hPDI with MetS, particularly abdominal obesity (206–208). Similar trends have also been reported in overweight or obese adolescents (209). Conversely, uPDI, characterized by refined carbohydrates, sugars, and high-salt foods, has been positively associated with MetS in Chinese and Korean populations (210, 211). It is noteworthy that in some obese populations, although the association between uPDI and overall MetS risk was not significant, it was closely related to a dramatic increase in hyperglycemia risk (OR ≈ 2.5) (212). Furthermore, this association exhibits significant population heterogeneity, with gender and age being important modifiers. Multiple studies point out that the protective effect of a healthy plant-based diet is more pronounced in women, particularly elderly women over 60 years of age (206, 208, 213).

#### 7.1.2 Effects on core components

PBD patterns exhibit specific and significant associations with individual components of MetS, which are also significantly modified by food quality. Regarding obesity and body fat distribution, a hPDI is significantly associated with an approximate 20% reduction in abdominal obesity risk (207), and plant protein intake has been shown to be superior to animal protein in reducing body fat and metabolic risk scores (214). In the realm of glucose metabolism, hPDI is associated with lower fasting glucose, insulin levels, and HOMA-IR index, while uPDI is closely linked to insulin resistance and hyperglycemia risk, especially in obese populations (212, 215), indicating that carbohydrate quality is a key variable determining glycemic outcomes. Regarding lipid profiles, healthy PBD significantly lower triglycerides and elevate HDL-C levels, whereas unhealthy patterns lead to hypertriglyceridemia and low HDL-C (211, 215, 216). Additionally, hPDI has been associated with lower blood pressure (208), and interventional evidence suggests that plant-based functional components such as phytosterols may contribute to vascular benefit (217). Overall, the metabolic effects of PBDs appear to depend more on the quality and processing of plant foods than on plant-food predominance alone.

#### 7.1.3 Potential biological mechanisms

The biological mechanisms by which PBD improve MetS involve the interaction between weight-dependent pathways and weight-independent metabolic regulation. First, mediation analysis shows that BMI reduction explains approximately 27.8% of the risk reduction in MetS by hPDI, indicating that weight control and reduced adiposity are foundational pathways for protection (207). Second, metabolomic analysis indicates that plant-based diets correlate with a distinct plasma profile, including lower levels of glutamate and isoleucine, which is associated with improved insulin sensitivity (218). Furthermore, plant bioactives, such as polyphenols and carotenoids, are suggested to modulate lipid metabolism and oxidative stress through pathways like AMPK activation or enhanced antioxidant enzyme activity (219, 220). Finally, animal studies suggest that dietary fibers and anthocyanins may modulate the gut microbiota and increase SCFA production, potentially supporting metabolic homeostasis (221).

### 7.2 PBD and T2DM

#### 7.2.1 Incidence risk and primary prevention

Epidemiological evidence widely supports the important role of high-quality plant-based diets in reducing the incidence risk of T2DM, but the distinction of dietary quality is paramount. Multiple large-scale cohort studies suggest that the hPDI shows a significant linear dose-response inverse correlation with the risk of T2DM and prediabetes, whereas the uPDI, rich in refined carbohydrates, is associated with increased incidence risk (222, 223). In a Korean cohort, each 10-point increase in hPDI was associated with an approximately 14% lower diabetes risk, particularly among individuals with a family history of diabetes or hypertension, whereas overall PDI and uPDI were not significantly associated with T2DM risk (224). Similar protective associations have also been reported for other high-quality plant-based patterns, including PHDI and the Portfolio Diet (225, 226). Notably, dietary intervention possesses cumulative and dynamic effects; increasing hPDI over time can reduce risk by 7%−9%, while a substantial decline in adherence is linked to a significant rebound in risk (227). These findings suggest that primary prevention should emphasize sustained intake of high-quality plant foods rather than simply reducing animal foods.

#### 7.2.2 Disease management and secondary prevention

Among patients with established type 2 diabetes, PBD interventions have been associated with lower HbA1c and BMI, and glycemic control effects superior to conventional non-vegetarian patterns (228, 229). Crucially, a high-quality PBD combined with moderate exercise as a comprehensive lifestyle intervention not only significantly reduces the use of hypoglycemic and cardiovascular medications but may also contribute to diabetes remission in some patients (230). Regarding long-term prognosis, higher hPDI has been associated with lower all-cause mortality in patients with T2DM or prediabetes, whereas unhealthy plant-based dietary patterns have been associated with increased mortality (231). This survival benefit may be more evident in patients with poorer metabolic status, including those with poor glycemic control, longer disease duration, larger WC, or earlier disease onset (232). Overall, current evidence suggests that the clinical relevance of PBDs in T2DM is closely related to dietary quality.

#### 7.2.3 Potential biological mechanisms

PBDs synergistically reduce T2DM risk through multiple biological pathways. First, weight loss is the primary mediating factor; high-fiber, low-calorie-density PBDs are associated with improved insulin sensitivity, a relationship largely mediated by reductions in BMI and WC (223, 227, 233). Second, weight-independent metabolic regulation mechanisms also play a role. High-quality PBD are rich in antioxidants and phytochemicals and are associated with lower levels of inflammatory biomarkers (223, 231). Additionally, PBDs are associated with increased richness and diversity of beneficial gut microbiota, potentially supporting improved glucose metabolism and insulin sensitivity (234).

### 7.3 PBD and CKD

#### 7.3.1 Incidence risk and primary prevention

Although systematic reviews suggest that PBD are generally associated with a lower risk of incident CKD (235), this general conclusion masks the heterogeneous effects of dietary composition. Large-scale studies based on the UK Biobank and Australian cohorts consistently emphasize that the potential renoprotective benefits of PBD are highly dependent on dietary quality. Only a hPDI, rich in whole grains, fruits, and vegetables, is significantly associated with reduced risk of incident CKD and improved eGFR; conversely, an uPDI, characterized by refined grains and sugars, not only such benefits but is positively correlated with increased risk (95, 236, 237). This preventive benefit is not limited to the general population but is equally significant in high-risk groups such as diabetics (238). Overall, current evidence suggests that the potential renal benefits of PBDs are more closely related to the quality of plant foods than to plant-based eating alone.

#### 7.3.2 Disease management and secondary prevention

In patients with established CKD, the prognostic associations of PBDs also depend on diet quality. Prospective studies from the UK Biobank and CRIC suggest that higher hPDI is associated with lower all-cause mortality, whereas higher uPDI is associated with worse prognosis (239, 240). NHANES data further suggested subgroup heterogeneity, with the adverse association of uPDI being more evident in women and in patients without diabetes; by contrast, overall PDI and hPDI did not show consistent independent associations (35). In clinical practice, hyperkalemia is often the main barrier preventing advanced CKD patients from adopting PBDs. However, a short-term feasibility trial in patients with stage 4–5 CKD and hyperkalemia suggested that, with potassium binders and close monitoring, a relatively high-potassium PBD can be implemented safely and may improve dietary fiber intake and quality of life (241). Nevertheless, broader implementation still requires balancing nutritional benefit against treatment cost and the potential risk of sarcopenia in older patients with severe restriction of animal protein (242).

#### 7.3.3 Potential biological mechanisms

The biological basis for improved kidney outcomes by PBD involves the synergistic regulation of molecular signals and systemic metabolism. First, evidence from animal models suggests that bioactive compounds like Fisetin may modulate the CD36/fibrosis pathway, potentially attenuating renal injury (243). Second, metabolomic research indicates that PBDs correlate with specific serum signatures, particularly those involving glucose and gamma-glutamyl peptide metabolism, which are associated with a lower risk of incident CKD (244). Finally, PBDs are associated with improved insulin sensitivity and reduced inflammatory markers like IL-6 and CRP, which may collectively support the preservation of renal function (245).

### 7.4 PBD and CVD

#### 7.4.1 Incidence risk and primary prevention

Although macro-level meta-analyses indicate that PBDs are associated with lower coronary heart disease mortality (246), their cardiovascular associations appear to depend largely on plant-food quality. Multiple studies show that hPDI is significantly inversely associated with CVD and mortality risk; conversely, uPDI is associated with increased risks (247–249). Furthermore, hPDI is significantly associated with lower risk of cardiometabolic multimorbidity, especially in individuals under 60, highlighting a potentially earlier window for dietary intervention (250). For vegetarian or vegan diets, evidence also supports benefits in reducing ischemic heart disease risk (251). These associations have also been reported across different genetic risk strata. Prospective studies show that hPDI is associated with lower cardiovascular risk in individuals with hereditary obesity and in those at higher genetic risk (252). Further research indicates that regardless of an individual's genetic risk score for myocardial infarction and stroke, hPDI is associated with reduced overall CVD incidence, supporting an association that appears independent of genetic susceptibility (253). In metabolically high-risk groups (e.g., prediabetes), the divergent effects of diet quality are further amplified: hPDI maintains protection, while uPDI is linked to higher CVD risk and adverse metabolic profiles (254). However, heterogeneity exists across demographic characteristics. While significant inverse associations were observed in multi-ethnic cohorts (255), no significant link between PDI, hPDI, or uPDI and CVD incidence was reported among African American populations in the Southern US (256). This discrepancy may be attributed to socioeconomic and food-environment differences that influence access to high-quality plant foods and cooking practices.

#### 7.4.2 Survival prognosis and secondary prevention

Compared to primary prevention, secondary prevention research in established CVD focuses on survival benefits. Cohort studies of CVD survivors show that higher overall PDI is associated with significantly lower all-cause mortality and shows a marginal inverse association with cardiovascular death; whereas higher uPDI is associated with increased risks of all-cause, cardiovascular, and cancer mortality (257). Additionally, PBD demonstrates potential in reversing pathological processes. Animal experiments suggest that PBD can not only prevent but even reverse hypertension-induced coronary microvascular dysfunction (258). This suggests PBD may serve as a potential therapeutic strategy for hypertension-related cardiovascular complications by improving myocardial microcirculation, although this requires validation in high-quality clinical trials.

#### 7.4.3 Potential biological mechanisms

Multi-omics technologies provide insights into the potential mechanisms linking PBDs to cardiovascular health. First, evidence from cross-sectional analysis suggests that chronic low-grade inflammation may act as a link between diet quality and cardiovascular prognosis; higher hPDI scores correlate with lower inflammatory markers like CRP and IL-6, whereas uPDI is associated with elevations in factors like complement C3 and TNF-α (259). Second, data from randomized controlled feeding trials indicate that PBDs may modulate metabolic profiles by increasing beneficial metabolites like N2-acetylornithine and reducing atherogenic lipids (260). Finally, large-scale proteomics have identified plasma proteins, including THBS2 and NT-proBNP, that are associated with PBD adherence and may serve as predictors of CVD subtypes like HF (261).

## 8 KD

**Figure 5:**
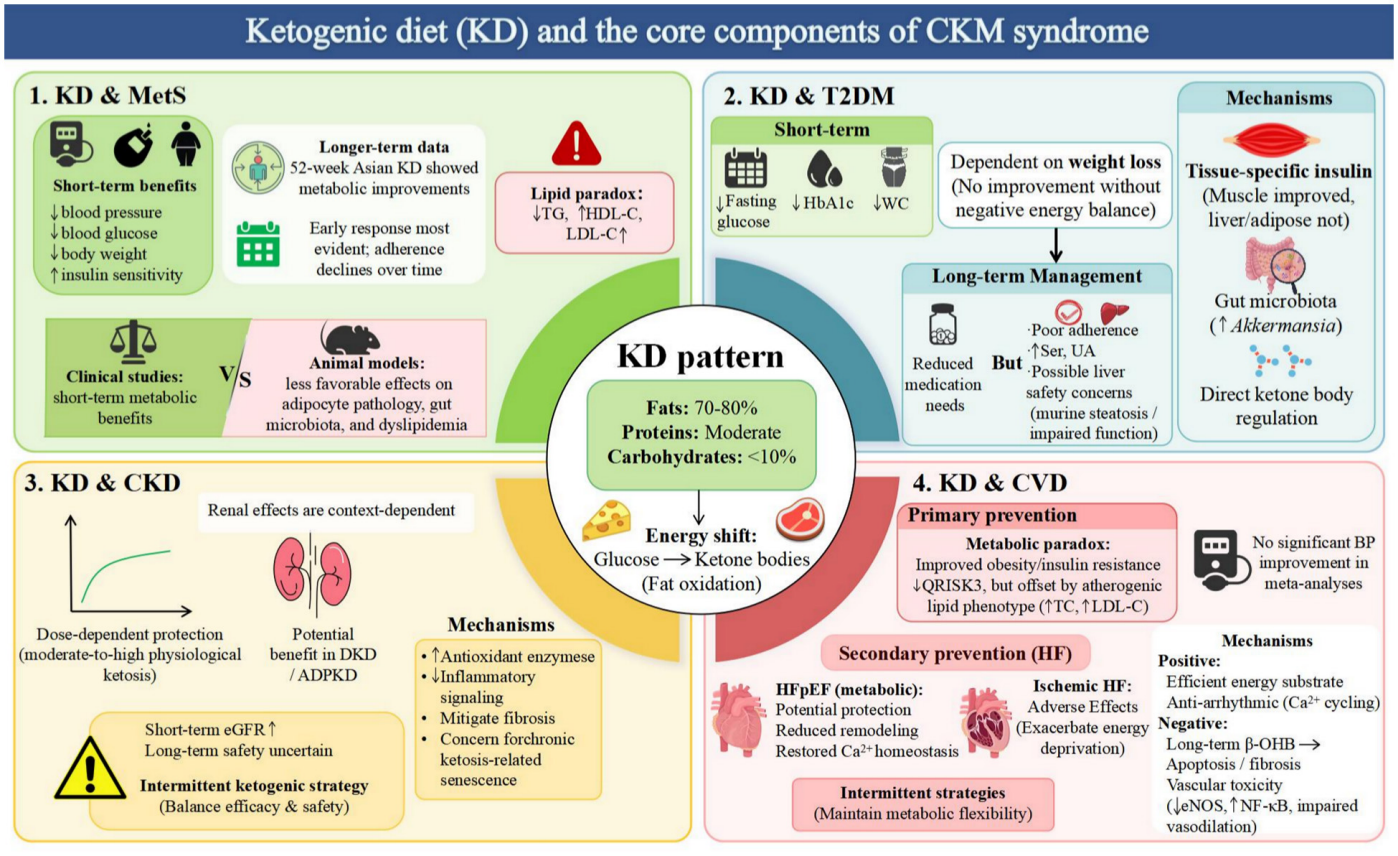
Ketogenic diet and the core components of CKM syndrome. KD may offer short-term metabolic benefits, including improved body weight, glucose control, TG, HDL-C, HbA1c, and WC, but its long-term role is limited by weight-loss dependence, LDL-C elevation, adherence issues, and safety concerns. Renal and cardiovascular effects appear context-dependent, with potential benefits in selected settings such as DKD, ADPKD, and HFpEF, but possible risks under chronic ketosis or ischemic HF. Potential mechanisms include energy substrate switching, β-OHB signaling, gut microbiota modulation, antioxidant defense, fibrosis regulation, calcium homeostasis, and endothelial effects. KD, ketogenic diet; CKM, cardiovascular-kidney-metabolic; MetS, metabolic syndrome; T2DM, type 2 diabetes mellitus; CKD, chronic kidney disease; CVD, cardiovascular disease; TG, triglycerides; HDL-C, high-density lipoprotein cholesterol; LDL-C, low-density lipoprotein cholesterol; HbA1c, glycated hemoglobin; WC, waist circumference; DKD, diabetic kidney disease; ADPKD, autosomal dominant polycystic kidney disease; HFpEF, heart failure with preserved ejection fraction; β-OHB, β-hydroxybutyrate.

The KD is a high-fat, moderate-protein, very-low-carbohydrate dietary pattern, typically providing 70%−80% of energy from fat and less than 10% from carbohydrates (262). Its potential effects on the core components of CKM syndrome are summarized in Figure 5.

### 8.1 KD and MetS

#### 8.1.1 Clinical evidence and intervention cycles

In the clinical management of MetS, the KD has shown favorable effects on body weight, glycemic control, and lipid metabolism. A meta-analysis of 13 RCTs showed that a very-low-carbohydrate ketogenic diet (VLCKD) was superior to low-fat diets in reducing body weight and diastolic blood pressure and in increasing HDL-C (263). These benefits appear most evident during the early phase of intervention. Hyde et al. showed that carbohydrate restriction was associated with rapid improvement in MetS markers within 4 weeks, with some lipid changes occurring independently of weight loss (264). Furthermore, a systematic evaluation of 14 RCTs in overweight individuals and patients with T2DM also supported benefits of KD on HbA1c, TG, and HDL-C (265). However, the effects of KD appear to be time-dependent. During the short-term phase (4–12 weeks), KD is generally associated with reductions in body weight, fasting insulin, and HbA1c, often with relatively good adherence (264, 266). With longer intervention, adherence tends to decline because of dietary restriction and poor sustainability (266). Despite these challenges, a 52-week study of the Asian KD reported significant improvements in adiposity, insulin sensitivity, lipid profile, and inflammatory markers, suggesting potential long-term metabolic benefits (267).

#### 8.1.2 Bidirectional effects on lipid profiles and synergistic therapeutic strategies

While KD is highly effective at lowering TG, its tendency to increase LDL-C remains a major concern (81, 268, 269). In a randomized trial comparing the MedDiet and KD, both diets reduced adiposity and improved insulin sensitivity, but the MedDiet showed more favorable LDL-C effects, whereas KD was associated with increased LDL-C (270). A 2024 RCT further suggested that a healthy KD with optimized fat quality may achieve weight loss without raising LDL-C, highlighting the importance of fat composition rather than carbohydrate restriction alone (271). However, animal studies have reported less favorable effects of KD on adipocyte pathology, gut microbiota, and dyslipidemia than balanced diets, suggesting that its metabolic effects may vary by model and context (272). Concurrently, KD has also been explored as an adjunctive strategy in obesity treatment. In patients receiving intragastric balloon therapy, VLCKD was associated with greater weight loss and metabolic improvement than conventional low-calorie diets (273). More recent evidence further suggests that, during treatment with weight-loss medications such as tirzepatide, ketogenic approaches may help preserve fat-free mass, muscle strength, and resting metabolic rate (274).

#### 8.1.3 Deep molecular mechanisms of gut microbiota and metabolic signaling

The metabolic effects of KD extend beyond substrate switching and may involve the gut microbiota, inflammatory signaling, and metabolic flexibility. In experimental studies, KD has been associated with changes in bile acid metabolism and gut microbial composition, which may contribute to improved glucose regulation (275). β-hydroxybutyrate, the major ketone body, has also been implicated in immune modulation, including suppression of Th17-related responses and inhibition of NLRP3 inflammasome activation (276, 277). However, these effects may not be uniformly beneficial. Some randomized trials found that KD reduced beneficial Bifidobacterium and was accompanied by higher hs-CRP, suggesting that immune and inflammatory responses may vary with diet composition or intervention duration (278).

KD may also enhance metabolic flexibility by shifting energy utilization from glucose to fat and ketone bodies, and experimental data suggest that this transition is associated with improved insulin sensitivity and mTOR inhibition (279). At the same time, early weight loss on KD should be interpreted cautiously, as part of the initial reduction may reflect loss of body water rather than adipose tissue (280). A 12-week feeding trial further showed that KD reduced fat mass while largely preserving resting metabolic rate and bone mineral density, although small changes in lean mass were partly attributable to alterations in body water (281).

### 8.2 KD and T2DM

#### 8.2.1 Short-term improvement of metabolic risk factors

In T2DM and prediabetes, KD has been associated with short-term improvements in glycemic control and central obesity. Multiple meta-analyses and clinical trials indicate that KD is associated with lower fasting glucose, HbA1c, and WC, with some studies suggesting greater glucose-lowering effects than the MedDiet and low-glycemic index diets (268, 282, 283). A cyclical KD intervention in newly diagnosed or overweight patients with T2DM also reported greater short-term metabolic improvement than conventional diabetic diets (269). However, these metabolic benefits appear highly dependent on weight loss. A strictly weight-controlled randomized intervention revealed that in the absence of weight loss, a low-carbohydrate KD did not significantly improve insulin sensitivity or glycemic control in T2DM patients, suggesting its metabolic dividend is primarily driven by negative energy balance rather than the direct effect of ketone bodies themselves (284).

#### 8.2.2 Long-term disease management and safety

In the long-term management of T2DM, KD has shown potential benefits for glycemic control, weight reduction, and medication de-escalation. A one-year prospective study reported that 60% of participants achieved diabetes reversal, defined as HbA1c < 6.5% without medications other than metformin (285). A 32-week RCT likewise showed that KD was associated with reductions in adiposity and use of antidiabetic medications (286), and digital coaching interventions have also been linked to improved HbA1c and reduced medication use over 5 months (287). However, these benefits may attenuate over longer follow-up. In a two-year study of veterans, the intervention group maintained lower BMI and reduced medication use, but no significant between-group difference in HbA1c was observed over 24 months (288). Furthermore, the endorsement of the KD as a lifelong dietary strategy is constrained by the inherent difficulty of long-term maintenance and the potential risk of elevated levels of serum uric acid (269). Safety also warrants attention. In murine models, a strict 9-month KD intervention limited weight gain but was also associated with hepatic steatosis, impaired liver function, and glucose intolerance (289). Taken together, KD may offer short- to medium-term metabolic benefits in T2DM, but its long-term sustainability and safety remain uncertain.

#### 8.2.3 Potential biological mechanisms

Potential mechanisms through which the KD may regulate glucose metabolism include tissue-specific shifts in insulin sensitivity, gut microbiota alterations, and direct ketone signaling. First, the tissue-specific divergence in insulin sensitivity is highly significant. Short-term KD intervention has been demonstrated to enhance insulin sensitivity within skeletal muscle, an effect primarily attributed to the augmentation of lipid utilization. However, the diet fails to induce comparable improvements in hepatic insulin sensitivity and may even diminish the inhibitory influence of insulin on adipose tissue lipolysis (290). Furthermore, emerging evidence indicates that ketogenic interventions may paradoxically impair systemic glucose tolerance. A 12-week randomized trial linked the KD to downregulated GLUT4 and upregulated PDK4 expression in skeletal muscle, suggesting reduced glucose disposal capacity (278). Additionally, the gut microbiota represents a potential mediator of these effects. Prospective research indicates that a VLCKD may enrich beneficial microbiota such as Akkermansia, correlating with metabolic improvements in diabesity (291). Finally, ketone bodies themselves may directly participate in glucose regulation through insulin-independent pathways (292).

### 8.3 KD and CKD

#### 8.3.1 Clinical efficacy and the potential for reversing metabolic derangements

In the prevention and treatment of CKD, particularly diabetic kidney disease (DKD), the KD has shown potential renoprotective associations. Evidence from multiple cohorts indicates that a higher ketogenic ratio in the diet is significantly associated with a reduced risk of ESKD. This protection is closely linked to circulating β-hydroxybutyrate (β-OHB) levels, with moderate-to-high physiological ketosis being associated with more favorable renal outcomes (293). Experimental work by Poplawski suggested that short-term ketogenic intervention may reverse inflammation- and fibrosis-related gene expression changes and improve albuminuria in diabetic nephropathy (294). Furthermore, the KD has shown clinical feasibility in the management of autosomal dominant polycystic kidney disease (ADPKD). The research of Torres found that BHB was associated with delayed renal cyst growth (295). Subsequently, the KETO-ADPKD randomized controlled trial provided the first human evidence that a 3-month ketogenic intervention was associated with a reduction in total kidney volume (296). Retrospective data further showed substantial weight loss with stable or slightly improved eGFR after 6 months of KD (297). Overall, current evidence suggests possible benefit in selected kidney disease settings, although clinical data remain limited.

#### 8.3.2 Potential biological mechanisms

The potential renoprotective effects of the KD may be mediated through enhanced antioxidant defense and attenuation of fibrotic remodeling. KD has been associated with reduced inflammatory signaling and increased antioxidant enzyme activity, changes that may help mitigate ischemia–reperfusion injury and renal fibrosis (298). Consistently, in unilateral ureteral obstruction models, KD alleviated interstitial fibrosis, possibly by limiting interstitial macrophage proliferation (299). However, the renal response to ketogenic intervention appears to depend on the underlying disease context. In spontaneously hypertensive rats, for example, a 4-week KD aggravated renal insufficiency and interstitial fibrosis, possibly through inhibition of autophagy and worsening of lipid metabolic disturbances (300). Therefore, these findings suggest that the renal effects of KD are context-dependent, and caution is warranted when extrapolating mechanistic observations to clinical practice, particularly in patients with kidney disease complicated by severe hypertension.

#### 8.3.3 Evolution of intervention strategies: from continuous to intermittent regimens

Although a very low-energy KD may induce a short-term increase in eGFR and offer metabolic benefits in patients with mild renal impairment, the safety of long-term continuous ketosis remains uncertain (301). Long-term intake of the KD may lead to gut microbiota dysbiosis and disrupt the metabolism of bile acids; abnormal levels of bile acids can interfere with core receptors in the liver, thereby inducing hyperketonemia and abnormal lipid accumulation in the liver (302). Additionally, evidence from animal models indicates that chronic sustained ketosis may provoke the p53-dependent senescence of renal cells (303). Based on these considerations, current clinical consensus increasingly favors an intermittent ketogenic strategy. Compared to continuous ketosis, an intermittent KD has been associated with more favorable mitochondrial metabolism, gut microbiota remodeling, and greater microbial diversity (304). Therefore, current evidence suggests that ketogenic interventions in kidney disease may require careful adjustment of treatment cycles to balance metabolic benefit against potential toxicity.

### 8.4 KD and CVD

#### 8.4.1 Incidence risk and primary prevention

At the primary prevention level, the KD exhibits a significant metabolic paradox. As a potent metabolic intervention, KD has been associated with improvements in obesity, insulin resistance, triglycerides, and QRISK3 cardiovascular risk scores in the short term (305, 306). However, these metabolic benefits are largely offset by its negative impact on the lipid profile. Due to high fat intake, KD often induces elevations in TC and LDL-C levels (307), and this shift toward an atherogenic lipid phenotype remains a major concern. Furthermore, KD lacks robust evidence for blood pressure control. Although some studies suggest benefits for blood pressure in obese populations (120, 308), high-quality meta-analyses indicate KD has no significant improvement effect on either systolic or diastolic blood pressure (309). Taken together, current evidence suggests that the role of KD in cardiovascular primary prevention should be interpreted cautiously, as short-term metabolic improvements may be offset by adverse lipid effects and limited antihypertensive benefit. Interestingly, long-term observations provide a more nuanced perspective on lipid-associated risks. Patients with type 1 diabetes who adhered to the KD for 10 years exhibited significantly elevated lipid levels; however, their coronary artery calcification scores remained extremely low (310). This finding adds nuance to the interpretation of lipid-related risk, but its clinical significance remains uncertain.

#### 8.4.2 Disease management and secondary prevention

In the secondary prevention of diseases like HF, the efficacy of KD appears to vary by etiology. Existing evidence suggests that KD has potential protective effects on HF with preserved ejection fraction characterized by metabolic disorders and diabetic cardiomyopathy. In addition to reducing left ventricular remodeling and lowering NT-proBNP levels, its key mechanisms involve delaying disease progression by restoring myocardial calcium homeostasis and electrophysiological function (311, 312). By contrast, KD may be less favorable in ischemic HF. Because the ischemic heart relies more heavily on glucose oxidation, KD-induced increases in free fatty acids and suppression of glucose utilization may aggravate myocardial energy deficiency and contribute to functional deterioration (313). Additionally, the mode of intervention may also modify these effects. Continuous ketosis has been associated with adaptive decline and a higher risk of fibrosis, whereas intermittent strategies such as the alternate-day ketogenic diet have shown more favorable effects on cardiac function and fibrosis (314). Taken together, current evidence suggests that in cardiovascular secondary prevention, the value of KD may depend not only on disease phenotype but also on how the diet is implemented.

#### 8.4.3 Potential biological mechanisms

The biological impact of KD on the cardiovascular system involves complex remodeling of energy metabolism, ion homeostasis, and signaling pathways. Regarding positive mechanisms, ketone bodies not only serve as efficient energy substrates to compensate for metabolic deficits but also exert anti-arrhythmic effects by elevating sarcoplasmic reticulum Ca^2+^ content, regulating calcium cycling proteins, and restoring Na^+^/ Ca^2+^ homeostasis, thereby improving myocardial diastolic function and shortening the QTc interval (311, 312). However, negative mechanisms are equally clear. Prolonged high-level exposure to β-OHB has been linked to cardiomyocyte apoptosis and fibrosis, possibly through inhibition of mitochondrial biogenesis and activation of SIRT7 signaling (315). At the vascular biology level, although KD may reduce plaque formation in some models by influencing macrophage polarization (316), it has also been suggested to impair endothelial function through reduced eNOS expression and activation of NF-κB signaling (317). Overall, current evidence suggests that the cardiovascular effects of KD are context-dependent and may differ across tissues and disease states.

## 9 Discussion

**Figure 6:**
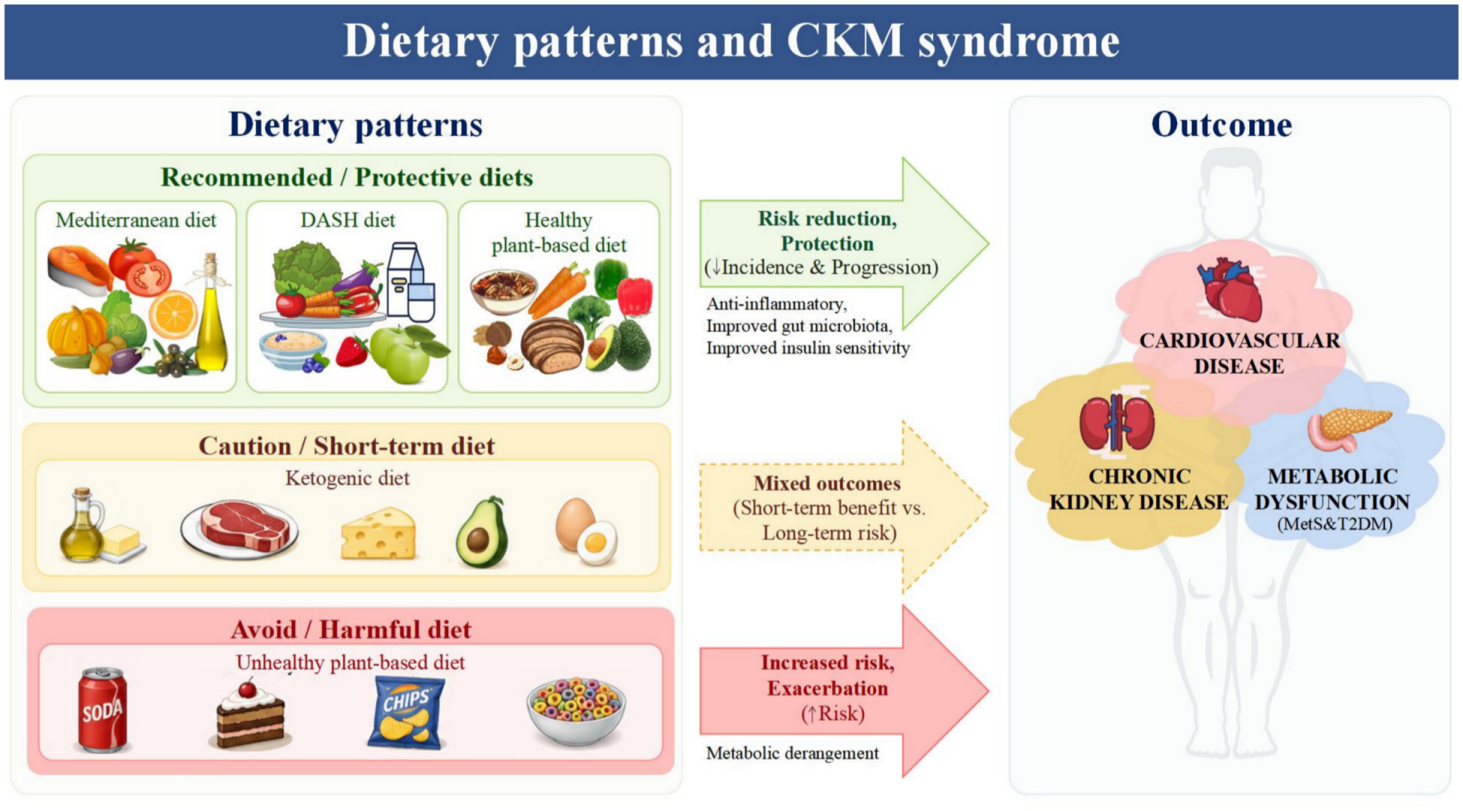
Comparative overview of major dietary patterns and their potential effects on CKM syndrome. The figure summarizes the potential roles of the Mediterranean diet (MedDiet), DASH diet, healthy plant-based dietary patterns, unhealthy plant-based dietary patterns, and ketogenic diet (KD) in relation to metabolic, kidney, and cardiovascular outcomes. MedDiet, DASH diet, and healthy plant-based dietary patterns are generally associated with favorable CKM-related outcomes, whereas unhealthy plant-based dietary patterns may be associated with increased CKM-related risk. The KD may provide short-term metabolic benefits, but its long-term role remains uncertain because of mixed evidence and potential safety concerns. CKM, cardiovascular-kidney-metabolic; DASH, dietary approaches to stop hypertension; KD, ketogenic diet.

Our review suggests that MedDiet and DASH diets tend to be regarded as foundational strategies for the early prevention of CKM syndrome. This potential may be attributed to their emphasis on high fiber, unsaturated fatty acids, and low sodium intake, which collectively show potential in synergistically improving blood pressure, lipid profiles, and insulin sensitivity (52, 53, 82, 143, 144, 148, 152). However, the generalizability of these dietary patterns across diverse ethnic and cultural backgrounds still warrants further validation. Regarding PBD, the clinical efficacy observed in this study appears to depend largely on dietary composition. Current evidence tends to suggest that only hPDI, characterized by whole grains, fruits, and vegetables, may yield positive prognostic outcomes. Conversely, excessive intake of refined carbohydrates or added sugars (uPDI) might offset the metabolic advantages typically associated with a PBD (205, 222, 223, 236, 237). This finding suggests that emphasizing dietary quality in clinical guidance may be more practical than simply focusing on the plant-based source itself. For the KD in the context of CKM syndrome, it could currently be positioned as a short-term clinical intervention, particularly for specific stages requiring rapid improvement in overweight or acute metabolic disturbances (264, 266, 269). Although some studies indicate potential benefits for glycemic control in the short term, its high fat content may have complex effects on long-term lipid profiles (268, 282, 283, 307). Given the relative lack of long-term safety data, clinical application should remain cautious. A comparative overview of these dietary patterns is provided in Figure 6.

Due to the nature of this narrative review, the breadth and depth of the evidence synthesis may have certain limitations. Notably, while this review prioritized systematic reviews and meta-analyses based on multiple RCTs, much of the summarized evidence is derived from observational studies, which inherently limits the ability to draw definitive causal conclusions. Furthermore, although significant associations have been observed between dietary patterns and outcomes across the various stages of CKM syndrome, these findings may be influenced by residual confounding, such as unmeasured lifestyle factors or socio-ecological variables. Additionally, the possibility of reverse causation, in which individuals alter their diet in response to a subclinical or diagnosed condition, cannot be entirely ruled out, particularly in cross-sectional analyses. Moreover, targeted research on the application of the KD across the stages of CKM syndrome remains notably insufficient in the current literature. Consequently, future research directions should focus on constructing more precise nutritional intervention frameworks. By exploring the molecular mechanisms through which different dietary patterns may block the progression of CKM stages, combined with individual metabolic characteristics, it is hoped that a more targeted evidence-based foundation for preventing CKM disease progression can be established.
