# Supplementary material for: A narrative review of dietary patterns in cardiovascular-kidney-metabolic syndrome
Source: Front Nutr. 2026 Jul 14;13:1799305. doi: 10.3389/fnut.2026.1799305 (PMC13407365; doi:10.3389/fnut.2026.1799305)
Supplement: Supplementary file 1 [file Data_Sheet_1.pdf]

Supplementary Table S1. Top 20 authors in dietary patterns and cardiometabolic–kidney health research.

Rank	Author	Documents	Citations	Average Citation/Publication
1	Salas-Salvado, J.	178	17483	98.22
2	Estruch, R.	142	15559	109.57
3	Corella, D.	128	13374	104.48
4	Ros, E.	121	13639	112.72
5	Hu, F. B.	118	18360	155.59
6	Martinez-Gonzalez, M. A.	116	9962	85.88
7	Fito, M.	99	5616	56.73
8	Serra-Majem, L.	86	10377	120.66
9	Ruiz-Canela, M.	86	4961	57.69
10	Toledo, E.	76	5978	78.66
11	Panagiotakos, D. B.	73	3588	49.15
12	Aros, F.	72	10275	142.71
13	Lopez-Miranda, J.	70	3297	47.1
14	Lapetra, J.	69	8964	129.91
15	Pinto, X.	67	7992	119.28
16	Perez-Martinez, P.	66	2734	41.42
17	Fiol, M.	65	8267	127.18
18	Gomez-Gracia, E.	64	8886	138.84
19	Delgado-Lista, J.	61	2812	46.1
20	Angel Martinez-Gonzalez, M.	55	7729	140.53

Average Citation/Publication was calculated as total citations divided by documents.

Supplementary Table S2. Top 20 journals in dietary patterns and cardiometabolic–kidney health research.

Rank	Source	Publications	Citations	Average Citation/Publication
1	Nutrients	867	33200	38.29
2	Frontiers in Nutrition	165	2503	15.17
3	American Journal of Clinical Nutrition	147	10484	71.32
4	Nutrition, Metabolism and Cardiovascular Diseases	121	5461	45.13
5	European Journal of Nutrition	109	3897	35.75
6	British Journal of Nutrition	105	4783	45.55
7	Journal of Nutrition	99	5366	54.20
8	Clinical Nutrition	81	2906	35.88
9	PLOS ONE	74	4166	56.30
10	International Journal of Molecular Sciences	70	4961	70.87
11	Public Health Nutrition	64	2384	37.25
12	Scientific Reports	59	856	14.51
13	European Journal of Clinical Nutrition	57	3333	58.47
14	International Journal of Environmental Research and Public Health	55	1723	31.33
15	Nutrition	54	2452	45.41
16	Advances in Nutrition	50	4592	91.84
17	International Journal of Food Sciences and Nutrition	50	1765	35.30
18	Antioxidants	50	1393	27.86
19	Nutrition Reviews	48	2202	45.88
20	Molecular Nutrition & Food Research	42	1457	34.69

Average Citation/Publication was calculated as citations divided by publications.

Supplementary Table S3. Top 20 countries in dietary patterns and cardiometabolic–kidney health research.

Rank	Country	Publications	Citations	Average Citation/Publication
1	United States	1840	118726	64.53
2	Spain	1180	65629	55.62
3	Italy	975	49458	50.73
4	China	507	18929	37.34
5	United Kingdom	439	30197	68.79
6	Australia	396	21960	55.45
7	Greece	350	16507	47.16
8	Canada	298	13970	46.88
9	Germany	263	18509	70.38
10	Iran	219	6737	30.76
11	France	192	10016	52.17
12	Netherlands	186	7881	42.37
13	Poland	160	6535	40.84
14	Sweden	155	9641	62.2
15	Brazil	152	5450	35.86
16	Denmark	105	5761	54.87
17	South Korea	105	2606	24.82
18	Japan	83	3045	36.69
19	Switzerland	74	3090	41.76
20	Norway	72	5630	78.19

Average Citation/Publication was calculated as citations divided by publications.
